# Integrated gut microbiome and serum metabolomics identify candidate metabolic signatures of uric acid nephropathy

**DOI:** 10.3389/fmicb.2026.1941602

**Published:** 2026-10-08

**Authors:** Min Cui, Sheng Li, Chenyu Wang, Jingxiu Dou, Yunyun Zhu, Qin Chen

**Affiliations:** College of Integrated Traditional Chinese and Western Medicine Clinical Medicine, Tongde Hospital of Zhejiang Province Affiliated to Zhejiang Chinese Medical University, Hangzhou, China

**Keywords:** 16s rDNA sequencing, hyperuricemia, metabolic signatures, serum untargeted metabolomics, uric acid nephropathy

## Abstract

**Objective:**

To characterize the clinical, gut microbial, and serum metabolic features of uric acid nephropathy (UAN) and identify candidate metabolic signatures distinguishing UAN from uncomplicated hyperuricemia (HUA).

**Methods:**

This observational study enrolled 108 patients with hyperuricemia, including 57 with HUA and 51 with UAN. Clinical characteristics, metabolic comorbidities, lipid profiles, and renal function indices were compared, and multivariable linear regression identified factors independently associated with estimated glomerular filtration rate (eGFR). Fecal 16S rRNA gene sequencing was performed in 72 participants (HC, *n* = 10; HUA, *n* = 30; UAN, *n* = 32), whereas serum untargeted metabolomic profiling was conducted in a nested subset of 30 participants (*n* = 10 per group). Differential taxa and metabolites were identified using LEfSe and combined multivariate and univariate analyses. Spearman correlation and MIMOSA2 analyses assessed microbiota–metabolite–clinical associations and predicted microbial contributions to candidate metabolites.

**Results:**

Patients with UAN were older and more frequently had hyperlipidemia than those with HUA. They also had higher serum creatinine, blood urea nitrogen, total cholesterol, low-density lipoprotein cholesterol, and urinary albumin-to-creatinine ratio, together with lower eGFR, despite comparable serum uric acid concentrations. Age and total cholesterol were independently associated with lower eGFR, whereas body mass index was positively associated with eGFR. Neither microbial α-diversity nor overall β-diversity differed significantly among groups. Exploratory microbiome analyses suggested higher *Faecalibacterium* abundance in HC, whereas selected taxa including *Streptococcus* and *Dialister* differed between HUA and UAN. OPLS-DA separated HUA from UAN, with 217 differential metabolites identified using VIP > 1.0 and *p* < 0.05. Exploratory multi-omics integration further highlighted nine candidate metabolic signatures. These metabolites were enriched mainly in glutathione metabolism, arginine and proline metabolism, histidine metabolism, glycerolipid metabolism, bile secretion, and ABC transporter pathways. Integrated analyses identified nine candidate metabolites: citric acid, succinic acid, deoxycytidine, uridine, guanosine, betaine, nicotinamide, glycerol, and stearic acid. Citric acid, deoxycytidine, succinic acid, and uridine showed the broadest predicted microbial contribution profiles.

**Conclusion:**

UAN was characterized by renal dysfunction, lipid metabolic abnormalities, selective gut microbial alterations, and extensive serum metabolic remodeling rather than by a further increase in circulating uric acid. The nine metabolites represent exploratory host–microbial metabolic signatures associated with hyperuricemia-related renal involvement. Their biological relevance and clinical discriminatory value require validation in larger, independent prospective cohorts.

## 1 Introduction

Hyperuricemia (HUA) is a common metabolic disorder defined by elevated serum uric acid levels and is largely driven by abnormalities in purine metabolism (Yanai et al., 2021). Alongside shifts in diet and lifestyle, the global prevalence of HUA has continued to rise (Zheng et al., 2020). In China, its overall prevalence is estimated at approximately 13.3%, placing HUA among the most frequent metabolic disorders after diabetes; notably, the age of onset has also shown a downward trend (Li et al., 2021; Zhang et al., 2021). Persistent hyperuricemia is linked not only to articular disease but also to kidney injury. Excessive uric acid deposition in the kidney can damage the tubulointerstitium and contribute to the development of uric acid nephropathy (UAN). Previous studies have reported renal impairment in nearly 40% of patients with HUA, while autopsy evidence has shown kidney involvement in a substantial proportion of patients with gout (Yang et al., 2025). Once established, renal injury is often difficult to reverse and may ultimately progress to end-stage kidney disease, creating a considerable clinical and socioeconomic burden. Early recognition and timely intervention in UAN are therefore of clear clinical importance.

The gut microbiota has received growing attention for its involvement in host metabolism, immune homeostasis, and preservation of intestinal barrier integrity (Liang et al., 2025; Qie et al., 2025; Zhang et al., 2025). Approximately one-third of uric acid is eliminated through the intestine. When gut microbial homeostasis is disrupted, opportunistic pathogens may expand, beneficial bacteria may decline, and intestinal barrier function may deteriorate. These changes can allow microbial metabolites to enter the circulation through the gut–kidney axis, activate inflammatory signaling, and worsen renal injury (Liu P. et al., 2025; Mo et al., 2026). Short-chain fatty acids (SCFAs), a major class of microbial metabolites, may facilitate uric acid excretion by supplying ATP, activating G protein-coupled receptors, and upregulating the urate transporter ATP-binding cassette subfamily G member 2 (Han et al., 2026; Wang Q. et al., 2025). In experimental models of UAN, SCFA-producing bacteria are markedly depleted, suggesting that microbial dysbiosis may promote disease progression through metabolite-dependent mechanisms (Lan et al., 2023).

Metabolomics offers a useful strategy for capturing the functional consequences of gut microbial alterations. Growing evidence indicates that HUA and UAN are closely associated with systemic metabolic disturbances, including excessive uric acid production and accumulation due to impaired purine metabolism (Liu F. et al., 2025), as well as lipid peroxidation, disrupted energy metabolism, and systemic inflammatory responses (Paduraru et al., 2025; Wu and You, 2023). Although HUA is accompanied by distinct changes in gut microbial composition and microbial metabolic functions, including pathways related to pyruvate metabolism, purine metabolism, and amino acid biosynthesis, the mechanisms by which gut microbiota dysbiosis reshapes host metabolic networks and contributes to renal injury remain incompletely understood (Martinez-Nava et al., 2025; Zhou et al., 2024). Current diagnostic strategies for UAN also have important limitations. Diagnosis based only on urinary findings or renal function indices lacks specificity and may result in misclassification, whereas renal biopsy, despite its greater diagnostic accuracy, is invasive and less acceptable to patients.

Against this background, the present study combined 16S rDNA sequencing, serum untargeted metabolomics, and clinical indicators to characterize gut microbial composition and serum metabolic profiles in healthy controls, patients with HUA, and patients with UAN. By integrating differential microbial taxa, metabolites, and their relationships with clinical phenotypes, we aimed to identify candidate metabolic signatures associated with renal involvement in hyperuricemia and to explore their potential discriminatory performance between UAN and uncomplicated HUA.

## 2 Methods

### 2.1 Study design

This cross-sectional observational study comprised two parts: a clinical cohort analysis and a multi-omics investigation. The study was conducted in accordance with the Declaration of Helsinki and the principles of Good Clinical Practice. Ethical approval was obtained from the Ethics Committee of Tongde Hospital of Zhejiang Province (Approval No. Zhejiang Tongde Ethics Review 2024, Research No. 176-JY). All participants provided written informed consent after receiving a detailed explanation of the study protocol.

Patients were recruited from the Department of Nephrology, Tongde Hospital of Zhejiang Province, between October 30, 2024, and October 30, 2025. All patients with hyperuricemia fulfilled the diagnostic criteria specified in the Chinese Guideline for the Diagnosis and Treatment of Hyperuricemia and Gout (2019). Under a normal purine diet, hyperuricemia was diagnosed when fasting serum uric acid levels exceeded 420 μmol/L on two separate days, irrespective of sex. For the clinical cohort, 108 patients with hyperuricemia were enrolled and divided according to the presence or absence of renal involvement into the hyperuricemia group (HUA, *n* = 57) and the uric acid nephropathy group (UAN, *n* = 51). Demographic characteristics, metabolic comorbidities, lipid profiles, and renal function-related indicators were collected and compared between the two groups. Multivariable regression analysis was then performed to identify clinical factors associated with estimated glomerular filtration rate (eGFR).

For the omics analyses, fecal 16S rRNA gene sequencing was performed in 72 participants, comprising 30 patients with HUA, 32 patients with UAN, and 10 healthy controls (HC). Healthy controls were recruited according to the predefined eligibility criteria; no formal individual or frequency matching was performed. Serum untargeted metabolomic profiling was conducted in a nested subset of 30 participants, comprising 10 individuals from each group. Thus, the microbiome and metabolomic analyses were performed in partially overlapping analytical cohorts rather than in an identical study population. Analyses integrating microbial and metabolic features were restricted to participants with paired data from both platforms.

Given the exploratory, hypothesis-generating nature of the omics analyses, no formal *a priori* sample-size calculation was performed, and the findings, particularly from the metabolomic and paired multi-omics analyses, require validation in independent cohorts.

Because the principal clinical comparison of the present study was between uncomplicated HUA and UAN, exploratory receiver operating characteristic (ROC) analyses were restricted to these two patient groups. Metabolite-based ROC analyses included the 20 patients with metabolomic data (HUA, *n* = 10; UAN, *n* = 10), whereas bacterial genus–based ROC analyses included all 62 patients with microbiome data (HUA, *n* = 30; UAN, *n* = 32). For direct comparison of the multivariable metabolic, microbial, and integrated models, analyses were restricted to the same paired-data subset of 20 patients (HUA, *n* = 10; UAN, *n* = 10).

### 2.2 Inclusion and exclusion criteria

For the clinical cohort, participants were classified into th HUA or UAN group according to prespecified eligibility criteria. Renal involvement was defined as UAN or other hyperuricemia-related renal abnormalities, including reduced eGFR, an increased urinary albumin-to-creatinine ratio, or other renal manifestations attributable to hyperuricemia after exclusion of major alternative kidney diseases. Hyperlipidemia was defined as a documented clinical diagnosis, current use of lipid-lowering medication, or an abnormal lipid profile characterized by elevated total cholesterol, triglycerides, or low-density lipoprotein cholesterol, and/or reduced high-density lipoprotein cholesterol, in accordance with established clinical criteria.

Patients were eligible for inclusion in the HUA group if they met all of the following criteria: (1) hyperuricemia diagnosed according to the Chinese Guidelines for the Diagnosis and Treatment of Hyperuricemia and Gout (2019), or a previous diagnosis of hyperuricemia without regular urate-lowering therapy during the preceding 3 months and without an acute gout flare at enrollment; (2) age 18–70 years; (3) complete clinical and laboratory data; and (4) provision of written informed consent. Exclusion criteria were secondary hyperuricemia attributable to malignancy, medication use, or another identifiable cause; an acute gout attack; definite UAN; gouty arthritis; tophi; hyperuricemia-related joint deformity or renal impairment; a psychiatric disorder; pregnancy or lactation; and incomplete clinical or examination data.

Patients were eligible for inclusion in the UAN group if they met all of the following criteria: (1) UAN diagnosed according to the Diagnostic Criteria, Syndrome Differentiation, and Therapeutic Evaluation of Uric Acid Nephropathy (Trial Version), issued by the Nephrology Branch of the China Association of Chinese Medicine; (2) age 18–70 years and the capacity to provide informed consent; (3) no regular use of urate-lowering medication during the preceding 3 months; (4) an eGFR of ≥15 and <90 mL/min/1.73 m^2^, calculated using the CKD-EPI-CysC equation; and (5) provision of written informed consent. Patients with electrolyte disturbances, acute infection, or other transient unstable conditions were enrolled only after clinical stabilization. Exclusion criteria were secondary hyperuricemia attributable to malignancy, medication use, or another identifiable cause; an acute gout attack; severe primary disease involving the nervous, cardiovascular, digestive, urinary, endocrine, or hematopoietic system; previous or ongoing renal replacement therapy; a psychiatric disorder; pregnancy or lactation; and incomplete clinical or examination data.

Participants with HUA or UAN who underwent multi-omics analysis were selected from the clinical cohort. Additional exclusion criteria were use of glucocorticoids, immunosuppressants, antibiotics, probiotics, or prebiotics within 2 months before sample collection; gastrointestinal disease, including gastroenteritis, during the preceding 2 months; and marked dietary restrictions or special dietary habits that could influence gut microbiota composition or serum metabolomic profiles.

Healthy controls were recruited according to the predefined eligibility criteria without formal individual or frequency matching to the patient groups. Eligible controls were 18–70 years of age, had no clinically detectable serious disease, and had normal serum uric acid concentrations, defined as <420 μmol/L in men and <360 μmol/L in women.

They were excluded if they had a psychiatric disorder; were pregnant or lactating; had used antibiotics, probiotics, or related preparations within 2 months before sample collection; had gastrointestinal disease within the preceding 2 months, including inflammatory bowel disease, chronic refractory constipation, active hemorrhoidal bleeding, or unexplained hematochezia; had undergone major abdominal surgery, such as gastrectomy; or had made substantial dietary changes within 1 week before sample collection.

### 2.3 Sample collection

Blood and fecal samples were collected from 30 patients with hyperuricemia, 32 patients with uric acid nephropathy, and 10 healthy controls who fulfilled the prespecified eligibility criteria. All participants were instructed to fast overnight for at least 8 h before sampling. Peripheral venous blood and fresh fecal samples were obtained in the morning.

Fresh fecal samples were placed in sterile, sealed collection containers within 2 h after defecation and immediately stored at −80 °C. For fecal sample preparation, 100 mg of frozen feces was diluted with a fivefold volume of sterile phosphate-buffered saline, vortexed for 1 min, and left to stand at 0 °C for 5 min. The suspension was subsequently centrifuged at 4 °C for 10 min. The resulting supernatant was collected, passed through a 0.22-μm sterile membrane, and stored at −80 °C until further analysis.

Blood samples were allowed to clot at room temperature for 1–2 h and then centrifuged at 3,000 rpm for 15 min. The serum supernatant was carefully collected and aliquoted to minimize repeated freeze–thaw cycles before being immediately stored at −80 °C for subsequent analysis.

### 2.4 Chemicals and instruments

Untargeted serum metabolomic profiling was conducted using ultra-high-performance liquid chromatography coupled with high-resolution mass spectrometry. Chromatographic-grade methanol, acetonitrile, and formic acid were used for sample preparation and chromatographic separation. The analytical platform consisted of a Vanquish ultra-high-performance liquid chromatography system coupled to a Q Exactive HF-X mass spectrometer (Thermo Scientific, Massachusetts, USA).

### 2.5 Serum metabolite extraction

Serum samples stored at −80 °C were thawed on ice until no visible ice crystals remained, and all subsequent procedures were carried out under low-temperature conditions. After thawing, each sample was vortexed for 10 s to ensure uniform mixing. A 50-μL aliquot of serum was transferred into a labeled centrifuge tube, followed by the addition of 300 μL of internal standard extraction solution containing 20% acetonitrile–methanol. The mixture was vortexed for 3 min and centrifuged at 12,000 rpm for 10 min at 4 °C. After centrifugation, 200 μL of the supernatant was transferred into a new labeled centrifuge tube and incubated at −20 °C for 30 min. The sample was then centrifuged again at 12,000 rpm for 3 min at 4 °C, and 180 μL of the final supernatant was transferred into an autosampler vial insert for liquid chromatography-mass spectrometry (LC–MS/MS) analysis.

### 2.6 LC–MS/MS conditions

Chromatographic separation was carried out on a Waters ACQUITY Premier HSS T3 column (1.8 μm, 2.1 mm × 100 mm). The mobile phases consisted of 0.1% formic acid in water for phase A and 0.1% formic acid in acetonitrile for phase B. The column temperature was maintained at 40 °C, with a flow rate of 0.4 mL/min and an injection volume of 3 μL. Gradient elution was programmed as follows: 5% B at 0.0 min, increasing to 20% B at 2.0 min, 60% B at 5.0 min, and 99% B at 6.0 min. The proportion of phase B was held at 99% until 7.5 min, then returned to 5% at 7.6 min, followed by equilibration until 10.0 min.

Mass spectrometric detection was conducted using an electrospray ionization source in both positive and negative ion modes. The ion spray voltage was set to 3,800 V in positive mode and 3,400 V in negative mode. The sheath gas and auxiliary gas were set at 60 Arb and 20 Arb, respectively. The ion transfer tube temperature was maintained at 320 °C, while the vaporizer temperature was set to 350 °C in positive mode and 650 °C in negative mode. Full MS scans were acquired across an m/z range of 70–1,000 at a resolution of 60,000, with an automatic gain control target of 3.00 × 10^6^. MS/MS scans were acquired over the same m/z range at a resolution of 15,000, with an automatic gain control target of 1.00 × 10^5^. Collision energies were set at 30, 40, and 50 V, with a stepped collision energy of 50 V. The intensity threshold was 1.00 × 10^6^ cps; the top 10 candidate ions were selected for fragmentation, and dynamic exclusion was set to 3 s.

### 2.7 Metabolomic data preprocessing

Raw mass spectrometry files were first converted to mzML format using ProteoWizard. Peak extraction, peak alignment, and retention time correction were then performed with XCMS. Features with missing values in more than 50% of samples were excluded. For the remaining features, missing values were imputed using either the K-nearest neighbor method or one-fifth of the minimum positive value, as appropriate. Peak areas were further adjusted by support vector regression to reduce systematic variation.

Metabolite annotation was conducted by matching the detected features against an in-house database, public databases, and predicted spectral libraries. Metabolites were retained for downstream analysis if they had an integrated identification score ≥ 0.5 and a coefficient of variation < 0.30 in quality control samples. Data acquired in positive and negative ion modes were subsequently merged. For duplicate annotations, the metabolite with the highest identification level and the lowest coefficient of variation was retained. A complete table of the annotated metabolites retained after quality-control and annotation filtering is provided in Supplementary Data 1.

### 2.8 Metabolomic statistical analysis

Multivariate and univariate analyses were conducted using R software. PCA was used to assess overall sample distribution and analytical stability, whereas OPLS-DA was used to explore metabolic separation between groups. Given the exploratory nature of the metabolomic analysis, metabolites with an OPLS-DA VIP score >1.0 and a nominal *p* value <0.05 were retained as the initial differential-metabolite screening set. To assess the impact of multiple testing, *p* values across all tested metabolites were additionally adjusted using the Benjamini–Hochberg false discovery rate (FDR) procedure; FDR-adjusted results were considered a sensitivity assessment rather than an additional screening criterion. Functional annotation and pathway enrichment analyses were subsequently performed using the KEGG and HMDB databases.

### 2.9 Fecal 16S rDNA sequencing and bioinformatic analysis

#### 2.9.1 Genomic DNA extraction and PCR amplification

Total genomic DNA was extracted from fecal samples using the CTAB/SDS method. DNA purity and concentration were assessed by agarose gel electrophoresis. Qualified DNA samples were diluted to 1 ng/μL with sterile water and used as templates for subsequent PCR amplification.

The V4 region of the bacterial 16S rDNA gene was amplified using barcode-tagged specific primers 515F and 806R. PCR amplification was carried out with Phusion® High-Fidelity PCR Master Mix with GC Buffer and high-fidelity DNA polymerase from New England Biolabs to ensure amplification specificity and accuracy.

#### 2.9.2 PCR product purification, library construction, and sequencing

PCR products were assessed by 2% agarose gel electrophoresis. Qualified amplicons were purified with magnetic beads, quantified, and pooled in equimolar proportions according to their concentrations. The pooled PCR products were then re-examined by 2% agarose gel electrophoresis, and the target bands were recovered using the Qiagen Gel Extraction Kit (Qiagen, Germany).

Sequencing libraries were prepared using the TruSeq® DNA PCR-Free Sample Preparation Kit. Library concentration and quality were evaluated using Qubit and quantitative PCR. Qualified libraries were subsequently sequenced on the Illumina NovaSeq 6,000 platform.

#### 2.9.3 Quality control and ASV generation

Raw sequencing data were quality-filtered to remove reads containing ambiguous bases; reads in which low-quality bases accounted for more than 40%; reads with a mean quality score <20 within a 4-base sliding window; reads containing terminal poly-G sequences; and reads shorter than 150 bp. High-quality paired-end reads were subsequently merged using FLASH. Chimeric sequences were identified and removed by comparison with the reference annotation database using VSEARCH, yielding high-quality clean data for downstream analysis.

Amplicon sequence variants were inferred from the clean data using the DADA2 denoising algorithm. For each ASV, the sequence with the highest frequency was selected as the representative sequence, and singleton sequences were removed to reduce sequencing noise. Venn or flower diagrams were then used to summarize shared and unique ASVs across samples and groups, allowing core and group-specific microbial features to be identified.

#### 2.9.4 Taxonomic annotation and microbial diversity analysis

Taxonomic annotation was conducted using the Mothur method against the SILVA database (version 138.1), with a confidence threshold set between 0.8 and 1.0. Taxonomic composition and abundance profiles were generated at the phylum, class, order, family, genus, and species levels. Relative abundance was defined as the proportion of sequences assigned to a given taxon relative to the total sequences in each sample.

Alpha diversity was evaluated using the phyloseq and vegan packages in R. Shannon, Simpson, Chao1, ACE, and Goods_coverage indices were calculated to characterize within-sample microbial richness and evenness. Rarefaction curves were generated to assess sequencing depth, and box plots were used to display between-group differences in alpha diversity. Statistical significance was determined using the Kruskal–Wallis test.

Beta diversity was assessed using principal coordinates analysis (PCoA) based on UniFrac distances and non-metric multidimensional scaling (NMDS) based on Bray–Curtis dissimilarity. Group differences in overall microbial community composition were further evaluated by permutational multivariate analysis of variance (PERMANOVA; Adonis) using Bray–Curtis dissimilarity, with R^2^ and *p* values reported. NMDS model fit was assessed using the stress value.

### 2.10 Differential taxa and functional prediction

Differential taxa were identified using linear discriminant analysis effect size (LEfSe) with an LDA score threshold of ≥4.0. An exploratory three-group analysis was performed across HC, HUA, and UAN, followed by a pairwise LEfSe analysis for the primary HUA–UAN comparison. Selected genera were further assessed using the Wilcoxon rank-sum test. For the HC-associated *Faecalibacterium* enrichment, *post hoc* HC–HUA and HC–UAN comparisons were performed using the Wilcoxon rank-sum test, with Cliff’s δ and 95% confidence intervals used to quantify effect size. Positive Cliff’s δ values indicated higher abundance in HC. *p* values for these two comparisons were adjusted using the Benjamini–Hochberg procedure.

To assess potential confounding in the HUA–UAN comparison, multivariable sensitivity analyses were performed. Genus-level microbial features and predicted KEGG pathway and KEGG Orthology (KO) profiles were modeled with disease group as the primary predictor and BMI as a continuous covariate (Model 1). For genus-level analyses, Model 2 further adjusted for lipid-lowering medication use at biospecimen collection. Urate-lowering therapy was not included because no participants were receiving regular urate-lowering treatment at sampling. HUA served as the reference group. Adjusted coefficients, 95% CIs, nominal *p* values, and Benjamini–Hochberg FDR-adjusted *p* values were reported. Genus-level analyses were additionally repeated after excluding lipid-lowering medication users. These sensitivity analyses complemented the primary exploratory LEfSe analysis.

### 2.11 Selection of key features and integrated correlation analysis

Key microbial and metabolic features for integrative analyses were prioritized using modality-specific criteria. Microbial taxa were selected from the exploratory three-group and HUA–UAN pairwise differential-abundance analyses and further evaluated according to taxonomic resolution, relative abundance, prevalence, consistency with genus-level nonparametric comparisons, biological relevance, and suitability for downstream integration. Formal differential-metabolite screening and exploratory multi-omics candidate prioritization were treated as distinct analytical steps: differential metabolites were defined according to the criteria described above, whereas candidate metabolites for integrative analyses were prioritized based on pathway context, microbiota–metabolite associations, MIMOSA2-predicted microbial contributions, and biological relevance to renal and metabolic phenotypes. This prioritization was exploratory and did not constitute a prespecified serial filtering procedure. Associations with clinical indices were subsequently evaluated using Spearman correlation as *post hoc* analyses and were not used for candidate selection; where multiple comparisons were formally evaluated, *p* values were adjusted using the Benjamini–Hochberg false discovery rate procedure. Correlation coefficients were visualized using clustered heatmaps, and an overview of the differential-metabolite analysis and subsequent exploratory multi-omics prioritization framework is provided in Supplementary Figure 10.

### 2.12 Statistical analysis

For the clinical cohort analysis, continuous variables were first assessed for normality using the Shapiro–Wilk test. Variables following a normal distribution were presented as mean ± standard deviation and compared with Student’s *t*-test, whereas non-normally distributed variables were summarized as median and interquartile range and compared using the Mann–Whitney U test. Categorical variables were reported as numbers and percentages and analyzed with the chi-square test or Fisher’s exact test, as appropriate. Because Albumin-to-Creatinine Ratio (ACR) showed a skewed distribution, it was log10-transformed as log_10_(ACR + 1) before analysis.

Multivariable linear regression was used to identify clinical factors associated with eGFR. eGFR was specified as the dependent variable, and clinically relevant variables or variables showing between-group differences in the univariate analysis were considered candidate independent variables. The final model included age, BMI, hyperlipidemia, and TC. Regression coefficients (β) and 95% confidence intervals were reported. Multicollinearity was evaluated using the variance inflation factor. Multiple-testing procedures for the metabolomic and integrative correlation analyses are described in Sections 2.8 and 2.11, respectively.

Exploratory ROC analyses were performed to evaluate the ability of selected metabolic and microbial features to distinguish UAN from uncomplicated HUA. Healthy controls were excluded because the clinically relevant comparison was between hyperuricemic patients with and without renal involvement. Univariate analyses included 20 patients with metabolomic data (10 per group) and 62 patients with microbiome data (HUA, *n* = 30; UAN, *n* = 32). Three multivariable models were evaluated: a metabolic model comprising citric acid, succinic acid, deoxycytidine, and uridine; a microbial model comprising *Dialister* and *Streptococcus*; and an integrated model combining all six features. For comparability, all multivariable models were assessed in the same paired-data subset (*n* = 20; 10 per group). Model performance was internally evaluated using out-of-fold predicted probabilities from repeated stratified 5-fold nested cross-validation. AUCs with 95% confidence intervals were reported, and optimal cutoffs were determined using the Youden index, with corresponding sensitivity and specificity.

## 3 Results

### 3.1 Clinical characteristics and renal–metabolic phenotypes of patients with hyperuricemia and uric acid nephropathy

A schematic overview of the study design and experimental workflow is presented in Figure 1. A total of 108 patients were included in the clinical analysis, comprising 57 patients with HUA group and 51 patients with UAN group. The baseline characteristics of the two groups are summarized in Table 1.

**Figure 1:**
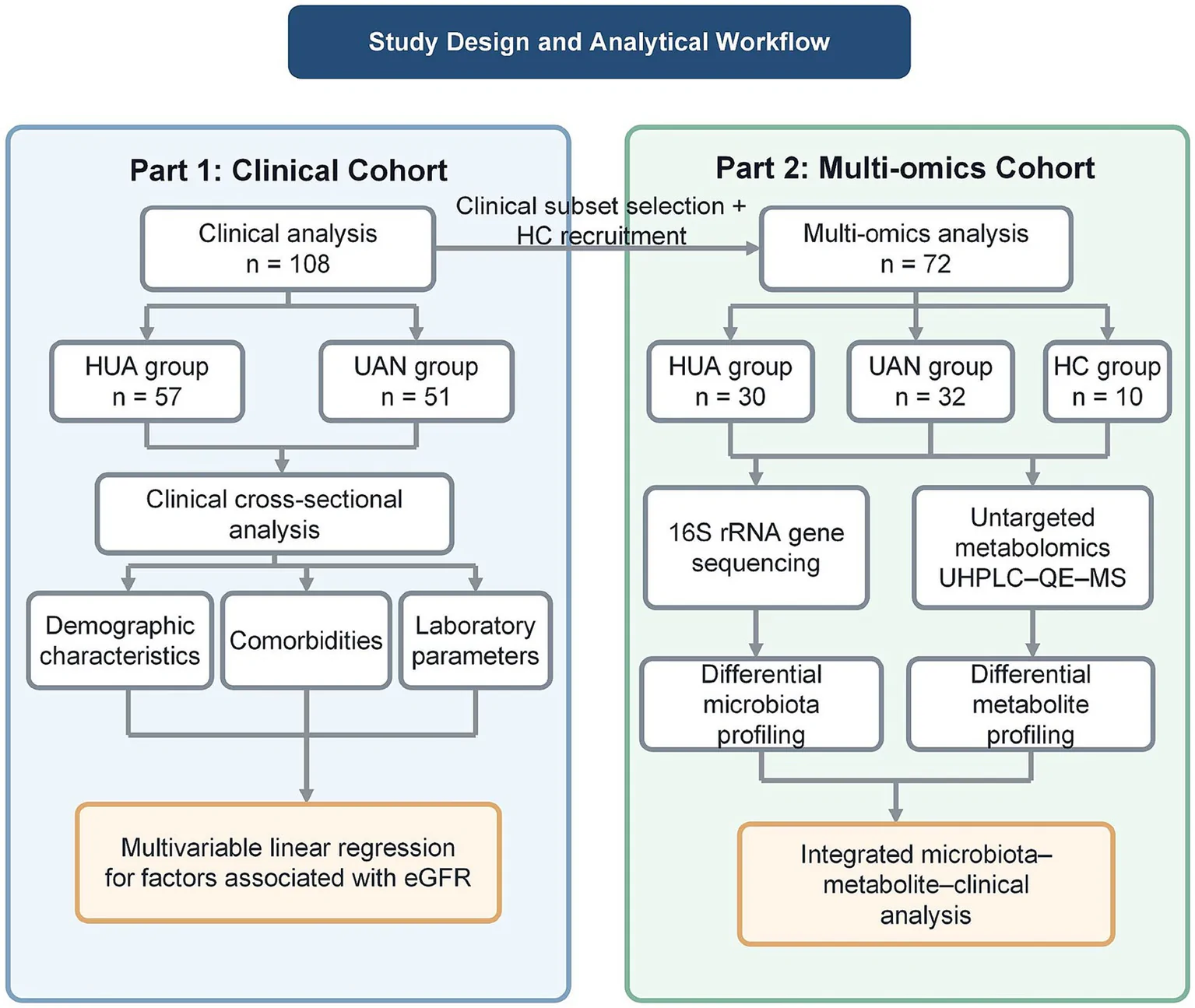
A schematic of the design and the experimental flow diagram.

**Table 1:** The general information and laboratory indicators of the HUA and UAN groups.

Characteristics	HUA group (*n* = 57)	UAN group (*n* = 51)	Test statistic	*p* value
Age (years)	36.12 ± 12.67	44.75 ± 15.29	*t* = −3.203	0.002*
BMI (kg/m^2^)	25.79 ± 2.77	24.09 ± 5.72	*t* = 1.991	0.049*
Smoking (*n*, %)	15 (26.32)	10 (19.61)	*χ^2^* = 0.681	0.409
Drinking (*n*, %)	13 (22.81)	10 (19.61)	*χ^2^* = 0.162	0.685
UA (μmol/L)	504.86 ± 140.24	498.20 ± 107.43	*t* = 0.275	0.784
Scr (μmol/L)	83.88 ± 10.07	116.04 ± 23.73	*t* = −9.340	<0.001*
BUN (mmol/L)	4.84 ± 1.27	5.67 ± 1.51	*t* = −3.117	0.002*
eGFR (ml/min)	103.27 ± 13.05	70.99 ± 13.14	*t* = 12.791	<0.001*
ALT (U/L)	24.00 (18.00, 36.00)	29.00 (18.00, 39.00)	*Z* = −0.951	0.345
TC (mmol/L)	4.90 ± 0.72	5.53 ± 0.95	*t* = −3.846	<0.001*
TG (mmol/L)	2.37 ± 0.99	2.67 ± 0.95	*t* = −1.598	0.113
HDL-C (mmol/L)	1.18 (1.04, 1.32)	1.18 (1.11, 1.26)	*Z* = −0.588	0.559
LDL-C (mmol/L)	3.20 ± 0.66	3.49 ± 0.75	*t* = −2.120	0.036*
ALB (g/L)	45.28 ± 2.41	44.45 ± 2.53	*t* = 1.748	0.083
ACR (mg/g)	20.10 (10.73, 29.30)	124.57 (8.45, 241.84)	*Z* = −3.939	<0.001*
Hypertension (*n*, %)	11 (19.30)	15 (29.41)	*χ^2^* = 1.512	0.220
Diabetes (*n*, %)	3 (5.26)	4 (7.84)	*χ^2^* = 0.021	0.879
Nephrolithiasis (*n*, %)	10 (17.54)	12 (23.53)	*χ^2^* = 0.595	0.441
Hyperlipidemia (*n*, %)	32 (56.14)	44 (86.27)	*χ^2^* = 11.721	<0.001*

HUA, hyperuricemia; UAN, uric acid nephropathy; Scr, serum creatinine; BUN, blood urea nitrogen; eGFR, estimated glomerular filtration rate; ACR, urinary albumin-to-creatinine ratio; UA, uric acid; TC, total cholesterol; TG, triglycerides; HDL-C, high-density lipoprotein cholesterol; LDL-C, low-density lipoprotein cholesterol; BMI, body mass index; ALT, Alanine aminotransferase; ALB, Albumin. *p < 0.05.

Age, body mass index, and the prevalence of hyperlipidemia differed significantly between the HUA and UAN groups (all *p* < 0.05). Patients in the UAN group were older than those in the HUA group, and body mass index showed a significant between-group difference (Figure 2C). Hyperlipidemia was also more common in patients with UAN (Figure 2B). In contrast, smoking history, alcohol consumption, and other comorbidities were comparable between the two groups (all *p* > 0.05).

**Figure 2:**
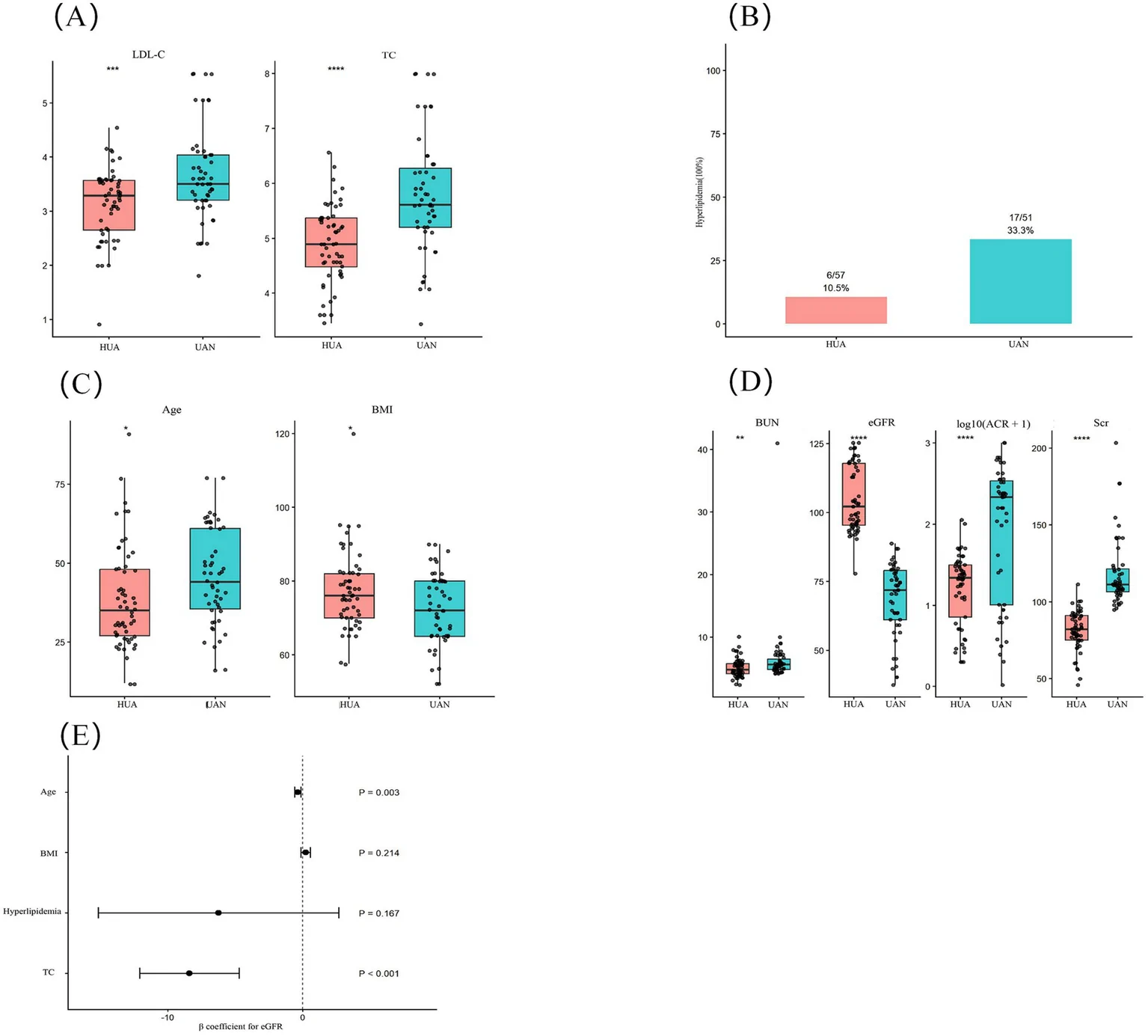
Clinical characteristics, lipid profiles, renal function parameters, and factors associated with eGFR in patients with HUA and UAN. **(A)** Comparison of serum LDL-C and TC levels between the HUA and UAN groups. **(B)** Prevalence of hyperlipidemia in the two groups; values above the bars indicate the number and proportion of patients with hyperlipidemia. **(C)** Comparison of age and BMI between the HUA and UAN groups. **(D)** Comparison of BUN, eGFR, log₁₀-transformed ACR [log₁₀(ACR + 1)], and Scr between the two groups. **(E)** Forest plot of standardized β coefficients and corresponding 95% confidence intervals from the multivariable linear regression analysis of factors independently associated with eGFR. HUA, hyperuricemia; UAN, uric acid nephropathy; LDL-C, low-density lipoprotein cholesterol; TC, total cholesterol; BMI, body mass index; BUN, blood urea nitrogen; eGFR, estimated glomerular filtration rate; ACR, urinary albumin-to-creatinine ratio; Scr, serum creatinine. *p < 0.05, **p < 0.01, ***p < 0.001, and ****p < 0.0001.

For laboratory indices, serum uric acid, alanine aminotransferase, high-density lipoprotein cholesterol, albumin, and triglyceride levels did not differ significantly between groups (all *p* > 0.05)(Figures 2A–D). Renal function-related parameters, however, showed a clear disease-associated pattern. Compared with patients in the HUA group, those in the UAN group had higher serum creatinine, blood urea nitrogen, and urinary albumin-to-creatinine ratio, together with a markedly lower eGFR (Figure 2D).

Several lipid metabolism-related parameters also differed between groups. Total cholesterol and low-density lipoprotein cholesterol were significantly higher in patients with UAN, whereas triglycerides, high-density lipoprotein cholesterol, alanine aminotransferase, serum albumin, and serum uric acid remained comparable between the two groups.

To identify clinical factors independently associated with renal function, we performed multivariable linear regression analysis using eGFR as the dependent variable. After adjustment for potential confounders, age, body mass index, and total cholesterol remained independently associated with eGFR (Figure 2E). Age and total cholesterol were negatively associated with eGFR, whereas body mass index showed a positive association with eGFR.

### 3.2 Gut microbiota alterations in hyperuricemia and uric acid nephropathy

As the clinical analysis identified renal dysfunction and lipid metabolic disturbance as key phenotypic features distinguishing UAN from HUA, indicators related to renal function and lipid metabolism were further included in the microbiota–clinical correlation analysis. Among the demographic and clinical variables, BMI differed significantly across the three groups (*p* = 0.020). By contrast, age, smoking status, alcohol consumption, and the prevalence of hypertension, diabetes mellitus, and nephrolithiasis were comparable among groups (all *p* > 0.05).

For laboratory parameters, significant between-group differences were observed in serum uric acid, serum creatinine, blood urea nitrogen, estimated glomerular filtration rate, total cholesterol, triglycerides, high-density lipoprotein cholesterol, and urinary albumin-to-creatinine ratio (all *p* < 0.05). Low-density lipoprotein cholesterol, alanine aminotransferase, and serum albumin did not differ significantly among the three groups (all *p* > 0.05). Overall, the three cohorts were broadly balanced in age, lifestyle factors, and major comorbidities, but showed clear differences in BMI and several biochemical indices related to uric acid metabolism, renal function, lipid metabolism, and urinary albumin excretion.

Given the potential influence of hyperuricemia and uric acid nephropathy on the intestinal microbiota, we next compared gut microbial diversity, community structure, taxonomic composition, and predicted functional profiles among the HC, HUA, and UAN groups. Sequencing depth was sufficient for downstream microbial community analysis, as both the species accumulation curve and sample-specific rarefaction curves progressively approached saturation with increasing sample size and sequencing depth (Supplementary Figure 1). Alpha diversity was assessed using the ACE, Chao1, Shannon, and Simpson indices. The HC group showed slightly higher median values for several indices, whereas the UAN group displayed greater inter-individual variability. None of the four α-diversity indices differed significantly among the HC, HUA, and UAN groups (all *p* > 0.05; Supplementary Figure 2). However, because the HC group comprised only 10 participants, these nonsignificant findings should not be interpreted as evidence of equivalence in microbial richness or within-sample diversity. The limited HC sample size reduced the statistical power to detect small-to-moderate between-group differences; therefore, the three-group diversity comparisons should be considered exploratory.

Beta-diversity analyses showed substantial overlap among the three groups. In the PCoA based on UniFrac distances, PC1 and PC2 explained 13.82 and 11.23% of the total variation, respectively, with HUA and UAN samples showing considerable overlap. PERMANOVA (Adonis) based on Bray–Curtis dissimilarity did not detect a significant difference in overall community composition among groups (R^2^ = 0.037, *p* = 0.054). NMDS showed a similar pattern without clear group separation, although the stress value was 0.20. Given the small HC sample (*n* = 10), these three-group β-diversity findings were considered exploratory (Figure 3A).

**Figure 3:**
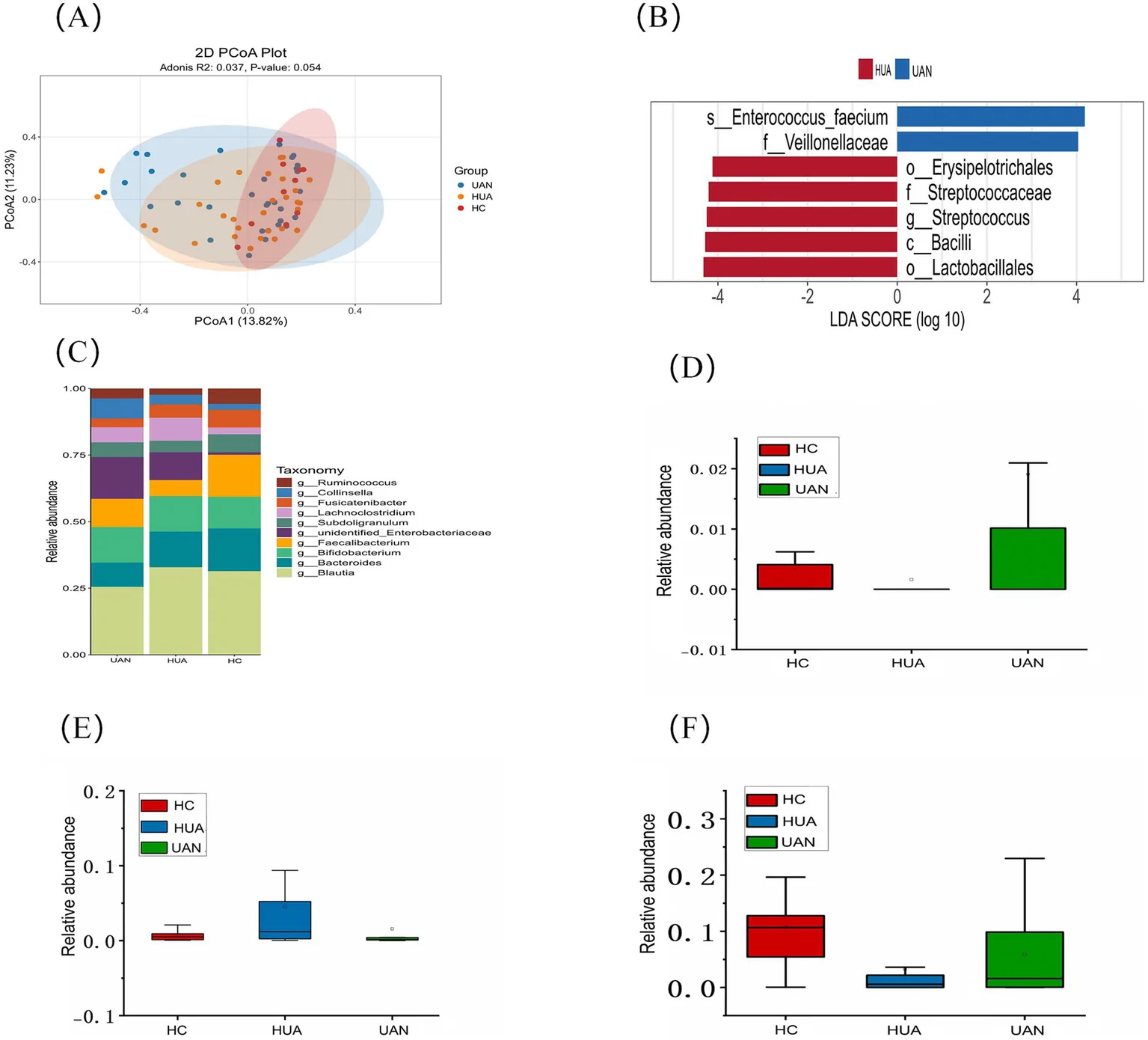
Alterations in gut microbial composition among the HC, HUA, and UAN groups. **(A)** Principal coordinates analysis (PCoA) based on UniFrac distances illustrating differences in overall gut microbial community structure among the HC, HUA, and UAN groups. Each point represents an individual sample, and the shaded ellipses indicate the distribution of samples within each group. Overall between-group differences were evaluated by PERMANOVA (Adonis; R^2^ = 0.037, *p* = 0.054). **(B)** Linear discriminant analysis effect size (LEfSe) analysis identifying differentially represented bacterial taxa between the HUA and UAN groups. Taxa enriched in HUA and UAN are shown in red and blue, respectively, with an LDA score threshold of ≥4.0. **(C)** Relative abundances of the predominant bacterial genera across the HC, HUA, and UAN groups. **(D–F)** Relative abundances of selected representative genera across the HC, HUA, and UAN groups, including *Dialister* **(D)**, *Streptococcus* **(E)**, and *Faecalibacterium* **(F)**. HC, healthy controls; HUA, hyperuricemia; UAN, uric acid nephropathy; PCoA, principal coordinate analysis; LDA, linear discriminant analysis; LEfSe, linear discriminant analysis effect size.

At the phylum level, the overall microbial composition was broadly similar across groups. Firmicutes was the dominant phylum in all three groups, followed by Bacteroidota, Proteobacteria, Actinobacteriota, and Verrucomicrobiota. Group-level stacked bar plots showed no obvious shift in the major phyla among groups. Sample-level profiles, however, revealed visible inter-individual variation, particularly in the relative abundances of Firmicutes, Bacteroidota, Proteobacteria, and Actinobacteriota, indicating heterogeneity in microbial composition across individuals.

At the genus level, the predominant genera included Blautia, Bacteroides, Bifidobacterium, Faecalibacterium, unidentified_Enterobacteriaceae, Subdoligranulum, Lachnoclostridium, Fusicatenibacter, Collinsella, and Ruminococcus. The HC, HUA, and UAN groups shared a broadly similar genus-level profile, suggesting partial consistency in the dominant microbial taxa. Still, the relative abundances of several genera varied across groups and individuals. Blautia, for example, accounted for a relatively large proportion in all groups, whereas Bacteroides, Bifidobacterium, Faecalibacterium, and unidentified_Enterobacteriaceae showed marked sample-to-sample variation. These findings indicate that genus-level differences were mainly reflected by shifts in the relative abundance of specific taxa, rather than by a complete restructuring of the dominant microbial community (Figure 3C). Detailed sample-level and group-level relative abundance profiles across the principal taxonomic ranks are provided in Supplementary Figure 3.

Exploratory three-group LEfSe analysis identified distinct group-associated bacterial taxa. *Faecalibacterium* was more abundant in HC; Bacilli, Lactobacillales, Streptococcaceae, *Streptococcus*, and Erysipelotrichales were enriched in HUA; and Veillonellaceae and *Dialister* were enriched in UAN (Supplementary Figures 4, 5A).

*Post hoc* pairwise analyses showed higher *Faecalibacterium* abundance in HC than in HUA (median [IQR], 0.1067 [0.0585–0.1271] vs. 0.0058 [0.0001–0.0211]; Cliff’s δ = 0.620, 95% CI 0.239–0.836; Wilcoxon *p* = 0.0038; BH-adjusted *p* = 0.0075). A similar direction was observed for HC versus UAN (0.1067 [0.0585–0.1271] vs. 0.0160 [0.0008–0.0958]; Cliff’s δ = 0.406, 95% CI 0.027–0.683), although this comparison was not statistically significant after BH correction (adjusted *p* = 0.0568) (Supplementary Table 1). Given the small HC sample size (*n* = 10), these comparisons were considered exploratory.

In the pairwise HUA–UAN LEfSe analysis, using an LDA score threshold of ≥4.0, Veillonellaceae and *Enterococcus faecium* were enriched in UAN, whereas Lactobacillales, Bacilli, Streptococcaceae, *Streptococcus*, and Erysipelotrichales were enriched in HUA (Figure 3B and Supplementary Figure 5B). Although *Dialister* did not reach the predefined LDA threshold in the pairwise analysis, direct genus-level comparison showed a higher abundance distribution in UAN than in HUA (Wilcoxon rank-sum test, *p* = 0.014; Cliff’s δ = 0.285, 95% CI, 0.060–0.483) (Supplementary Table 1). The relative abundances of *Dialister*, *Streptococcus*, and *Faecalibacterium* across the HC, HUA, and UAN groups are shown in Figures 3D–F.

After defining the taxonomic profiles, we compared the predicted microbial functional profiles among the three groups. Hierarchical clustering of differential KO terms showed that several functional units differed in relative abundance across the HC, HUA, and UAN groups. Compared with the HC group, K02026, K03088, and K03497 showed relatively lower abundance in the HUA and UAN groups, whereas K03657, K07024, and K00615 showed relatively higher abundance in the patient groups. These results suggest that, although the overall microbial community structure was not clearly separated among groups, the predicted functional composition displayed group-related differences.

Hierarchical clustering of differential EC terms showed a similar pattern. Several EC categories exhibited distinct relative abundance profiles among the three groups. EC:3.2.1.23, EC:2.7.7.7, and EC:3.4.21.89 were relatively more abundant in the HC group, whereas their abundance tended to be lower in the HUA and UAN groups. Conversely, EC:2.2.1.1, EC:3.6.3.17, and EC:2.7.1.69 showed relatively higher abundance in the patient groups. These findings indicate that hyperuricemia and uric acid nephropathy may be associated with alterations in microbiota-derived metabolic enzyme profiles.

KEGG functional classification further revealed group differences in several metabolism- and information-processing-related pathways. At KEGG level 2, lipid metabolism, amino acid metabolism, and metabolism of cofactors and vitamins were relatively enriched in the HC group and showed lower abundance in the HUA and UAN groups. In contrast, predicted functions related to endocrine and metabolic diseases and the immune system were relatively more abundant in the UAN group. Membrane transport and signal transduction also tended to be more abundant in the patient groups, particularly in the UAN group.

At KEGG level 3, purine metabolism, pyrimidine metabolism, and several amino acid metabolism-related functions were relatively enriched in the HC group but were reduced in the HUA and UAN groups. Conversely, glycolysis/gluconeogenesis, ABC transporters, and secretion system-related functions showed relatively higher abundance in the UAN group. Overall, exploratory KEGG functional profiling suggested group-related differences in several metabolism-, transport-, and signaling-related pathways. These signals were attenuated after adjustment for BMI and did not remain significant after FDR correction, indicating that potential BMI-related confounding cannot be excluded. The functional findings should therefore be considered hypothesis-generating and require validation using direct, higher-resolution functional approaches. Exploratory correlation analysis showed that the relative abundance of *Streptococcus* was positively associated with eGFR (*p* < 0.05; Figure 4). This association should be interpreted cautiously and requires validation in an independent cohort.

**Figure 4:**
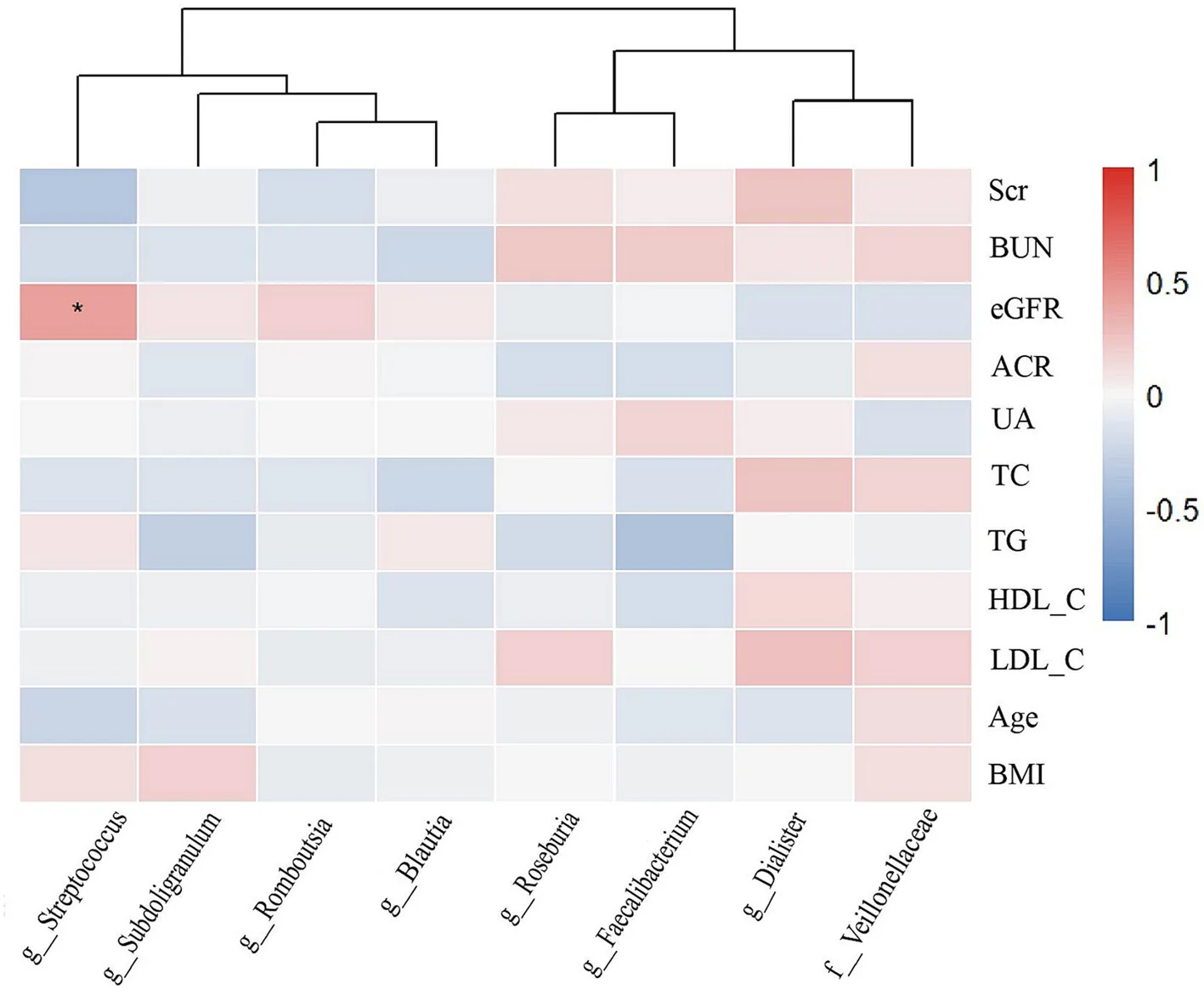
Associations between gut microbial taxa and clinical parameters in patients with hyperuricemia and uric acid nephropathy. Spearman’s correlation analysis was performed to evaluate the relationships between selected bacterial taxa and clinical indices. The color scale represents Spearman’s correlation coefficient, with red indicating positive correlations and blue indicating negative correlations. Bacterial taxa were hierarchically clustered according to the similarity of their correlation profiles. *p < 0.05. Scr, serum creatinine; BUN, blood urea nitrogen; eGFR, estimated glomerular filtration rate; ACR, urinary albumin-to-creatinine ratio; UA, uric acid; TC, total cholesterol; TG, triglycerides; HDL-C, high-density lipoprotein cholesterol; LDL-C, low-density lipoprotein cholesterol; BMI, body mass index; g__, genus; f__, family.

Medication use at biospecimen collection was summarized in Supplementary Table 5. Lipid-lowering therapy was more common in the UAN group than in the HUA group (40.6% vs. 10.0%, *p* = 0.008), whereas the use of statins, antihypertensive agents, ACEIs/ARBs, and glucose-lowering medications was similar between groups. No participants in either group were receiving regular urate-lowering therapy at the time of sampling. After adjustment for BMI (Model 1), *Streptococcus* abundance was lower and *Dialister* abundance was higher in UAN than in HUA. These directions were preserved after further adjustment for lipid-lowering medication use (Model 2). In Model 2, the adjusted coefficient was −2.135 for *Streptococcus* (95% CI, −3.583 to −0.687; nominal *p* = 0.005; FDR-adjusted *p* = 0.490) and 2.479 for *Dialister* (95% CI, 0.174–4.783; nominal *p* = 0.039; FDR-adjusted *p* = 0.986). Neither association remained significant after FDR correction. Analyses restricted to participants not receiving lipid-lowering therapy yielded similar directions of effect (Supplementary Table 5; Supplementary Data 3). Predicted KEGG pathway and KO associations also did not remain significant after BMI adjustment and FDR correction (Supplementary Table 4; Supplementary Data 2).

### 3.3 Serum metabolomic signatures across healthy controls, hyperuricemia, and uric acid nephropathy

Serum untargeted metabolomic profiling was performed in a nested subset of 30 participants, comprising 10 participants each from the HC, HUA, and UAN groups. Given the small sample size (*n* = 10 per group), HC-based comparisons were considered exploratory. To evaluate the stability and reliability of the metabolomics data, we performed correlation analysis using quality control (QC) samples. In both positive and negative ion modes, QC samples showed high correlation coefficients, indicating limited systematic variation, stable instrumental performance, and good reproducibility across sample preparation and data acquisition (Supplementary Figure 6). These findings confirmed the robustness and reliability of the metabolomics dataset.

Principal component analysis (PCA) was then used to examine the intrinsic clustering structure of the samples. In positive-ion mode, PC1 and PC2 explained 15.28 and 8.53% of the total variance, respectively; the corresponding values in negative-ion mode were 16.23 and 7.62%. The HUA and UAN groups largely overlapped, suggesting broadly similar serum metabolic profiles. By comparison, the HC group showed partial separation from the disease groups, although overlap was still present. The tight clustering of QC samples further supported the analytical stability of the platform (Figures 5A,B).

**Figure 5:**
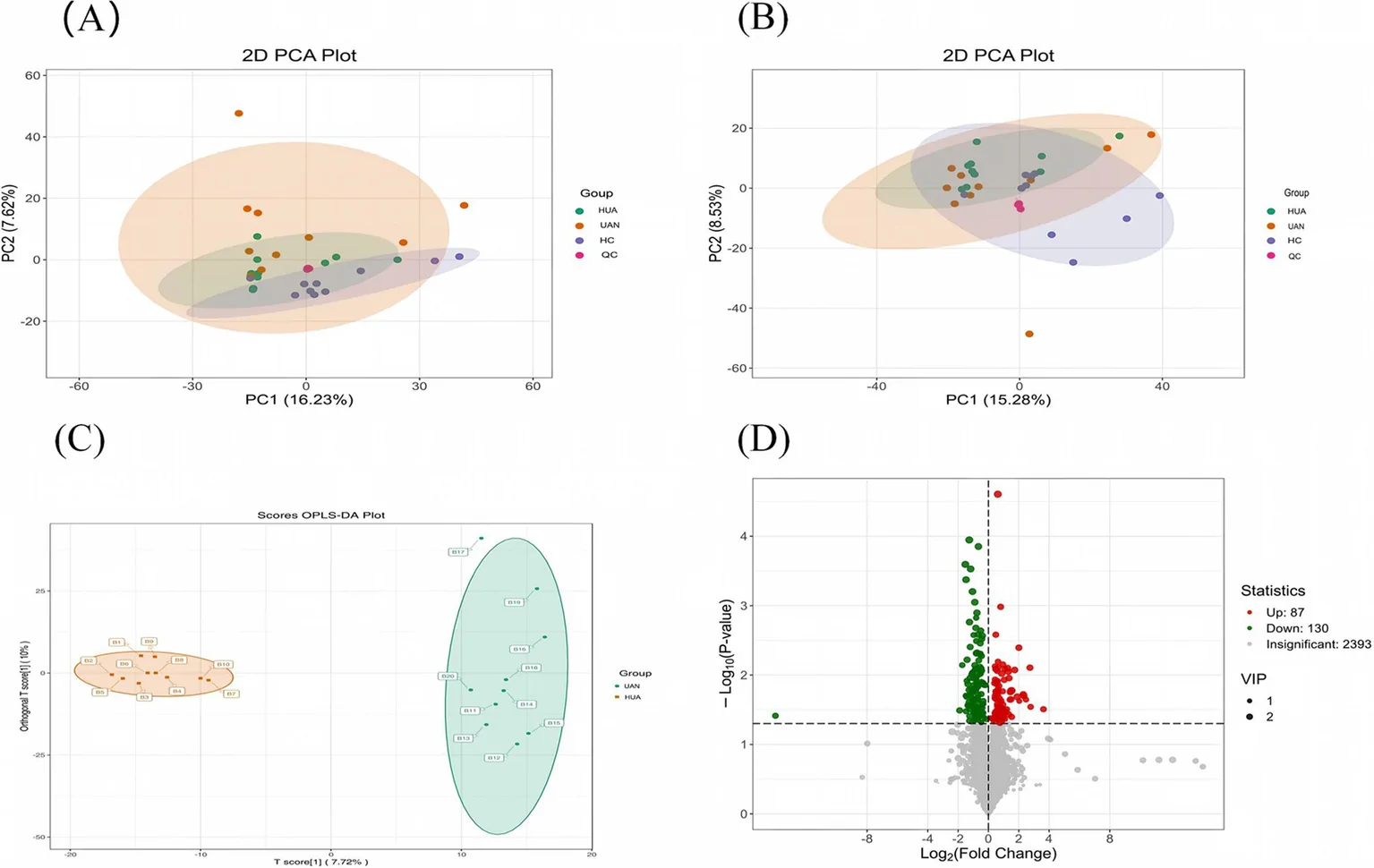
Serum metabolomic profiling and identification of differential metabolites in patients with hyperuricemia and uric acid nephropathy. **(A,B)** PCA score plots of serum metabolic profiles acquired in positive- and negative-ion modes, respectively. Each point represents an individual sample, and the shaded ellipses indicate the distribution of samples within each group. Pooled QC samples were included to evaluate the stability and reproducibility of the analytical platform. **(C)** OPLS-DA score plot illustrating the separation of serum metabolic profiles between the HUA and UAN groups. **(D)** Volcano plot showing differential metabolites between the HUA and UAN groups. Red and green points indicate significantly increased and decreased metabolites, respectively, whereas gray points represent metabolites without significant differences. Point size is proportional to the variable importance in projection (VIP) value. Differential metabolites were identified using VIP > 1 and *p* < 0.05. HC, healthy controls; HUA, hyperuricemia; UAN, uric acid nephropathy; QC, quality control; PCA, principal component analysis; OPLS-DA, orthogonal partial least squares–discriminant analysis; VIP, variable importance in projection.

Supervised orthogonal partial least squares discriminant analysis (OPLS-DA) showed a clear distinction between the HUA and UAN groups, which formed discrete clusters in the score plot (Figure 5C). This separation reflects differences in serum metabolic profiles between patients with HUA and those with UAN and suggests that renal involvement is accompanied by additional metabolic alterations. Model validation yielded R^2^X = 0.224, R^2^Y = 0.922, and Q^2^ = 0.581. Permutation testing showed no obvious overfitting, supporting the stability of the observed group discrimination of the OPLS-DA model (Supplementary Figure 7).

Using the exploratory criteria of VIP > 1.0 and nominal *p* < 0.05, 354 metabolites were identified in the HUA versus HC comparison, including 182 increased and 172 decreased metabolites, while 280 were identified in the UAN versus HC comparison, including 152 increased and 128 decreased metabolites. In the UAN versus HUA comparison, 217 metabolites met these criteria, of which 87 were increased and 130 were decreased (Figure 5D). These findings indicated differential serum metabolic patterns across the three groups, with additional alterations observed in UAN relative to HUA. However, none of the metabolites remained significant at an FDR threshold of <0.05 after Benjamini–Hochberg correction, and these findings were therefore considered exploratory rather than multiplicity-controlled discoveries.

To investigate the intrinsic relationships among differential metabolites, we constructed a metabolite correlation network based on correlation coefficients. Lipids and lipid-like molecules, especially glycerophospholipids and fatty acyls, together with organic acids and their derivatives, including keto acids and carboxylic acids, occupied central positions in the network. These metabolites may therefore represent key nodes involved in metabolic dysregulation associated with HUA and UAN.

Differential metabolites identified between the UAN and HUA groups were primarily enriched in pathways involved in redox homeostasis, amino acid and lipid metabolism, and membrane transport. The most prominently affected pathways included glutathione metabolism, arginine and proline metabolism, arginine biosynthesis, histidine metabolism, glycerolipid metabolism, bile secretion, and ABC transporters (Figure 6A). The KEGG regulatory network revealed extensive interconnections among metabolites, reactions, enzymes, and metabolic pathways, suggesting that the metabolic differences between UAN and HUA reflected coordinated perturbations across multiple biological processes rather than an isolated alteration in a single pathway (Figure 6B).

**Figure 6:**
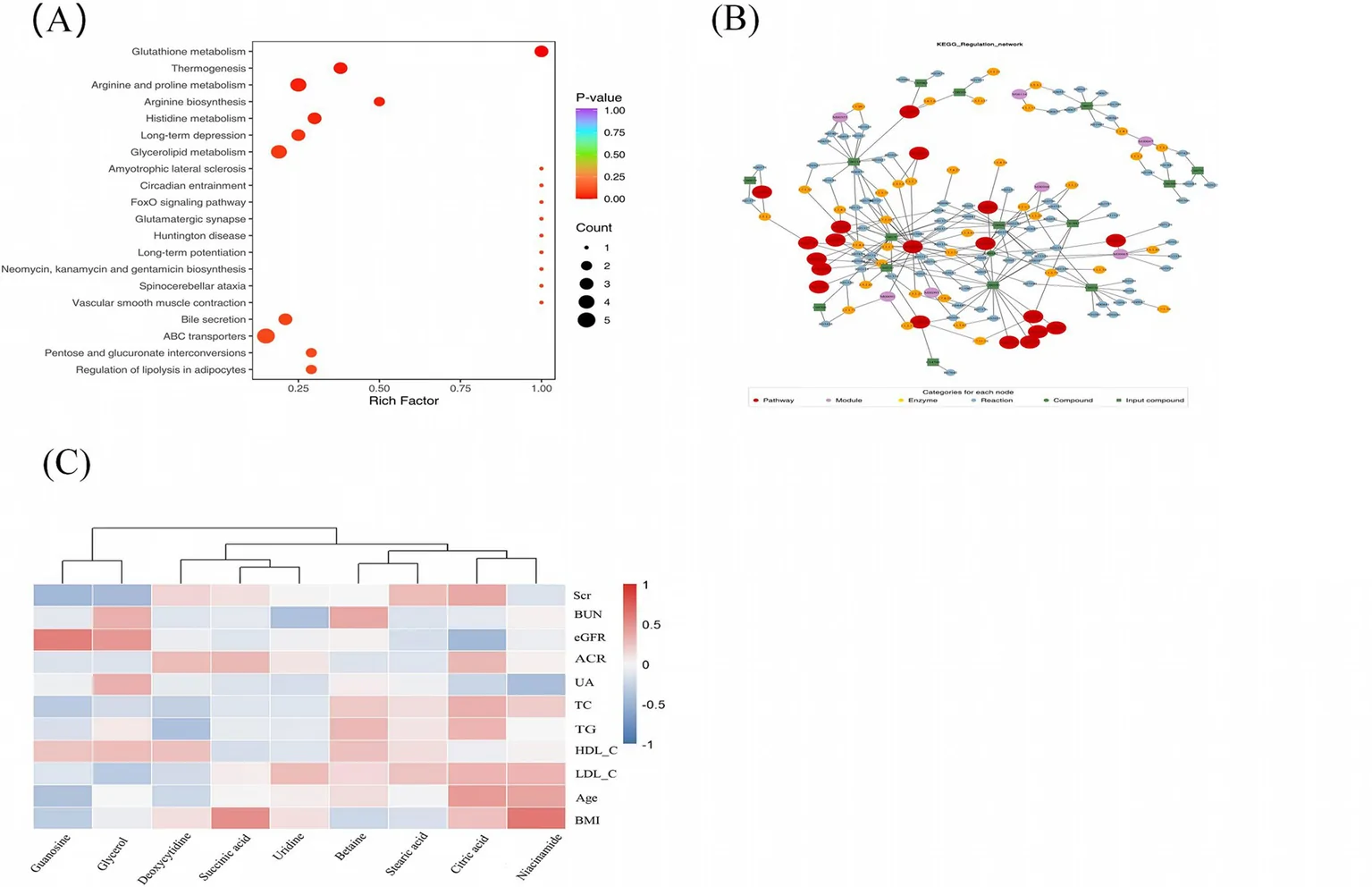
Functional enrichment and clinical associations of differential serum metabolites between patients with hyperuricemia and uric acid nephropathy. **(A)** Kyoto Encyclopedia of Genes and Genomes (KEGG) pathway enrichment analysis of differential metabolites between the HUA and UAN groups. **(B)** KEGG-based network depicting the relationships among enriched pathways, functional modules, enzymes, biochemical reactions, and associated metabolites. **(C)** Spearman correlation heatmap showing the associations between nine candidate metabolites—guanosine, glycerol, deoxycytidine, succinic acid, uridine, betaine, stearic acid, citric acid, and niacinamide—and clinical parameters. Red and blue indicate positive and negative correlations, respectively. Metabolites were hierarchically clustered according to the similarity of their correlation patterns. HUA, hyperuricemia; UAN, uric acid nephropathy; KEGG, Kyoto Encyclopedia of Genes and Genomes; Scr, serum creatinine; BUN, blood urea nitrogen; eGFR, estimated glomerular filtration rate; ACR, urinary albumin-to-creatinine ratio; UA, uric acid; TC, total cholesterol; TG, triglycerides; HDL-C, high-density lipoprotein cholesterol; LDL-C, low-density lipoprotein cholesterol; BMI, body mass index.

Correlation analysis demonstrated distinct associations between the nine candidate metabolites and clinical parameters related to renal function, uric acid metabolism, lipid metabolism, age, and BMI (Figure 6C). Guanosine and glycerol were positively correlated with eGFR, whereas citric acid was inversely correlated with eGFR and positively associated with ACR and several lipid-related indices. Collectively, these results link the identified metabolic alterations to impaired renal function and disrupted lipid metabolism in UAN.

### 3.4 Integrated microbiota–metabolite– clinical correlation analysis

We then analyzed the correlations between core differential taxa and candidate metabolites, which revealed marked microbiota–metabolite associations (Figure 7). The correlated metabolites were mainly involved in short-chain fatty acid-related metabolism, nucleoside metabolism, lipid metabolism, and inflammation-related metabolic processes. Together, these findings indicate associations between the observed gut microbial differences and serum metabolic alterations in HUA and UAN. MIMOSA2 analysis provided additional model-based evidence consistent with a possible microbial component of the observed metabolic patterns. Predicted contributions from multiple microbial taxa were identified for all nine candidate metabolites. Citric acid, deoxycytidine, succinic acid, and uridine showed the broadest microbial contribution profiles, with 59, 46, 44, and 35 contributing species, respectively.

**Figure 7:**
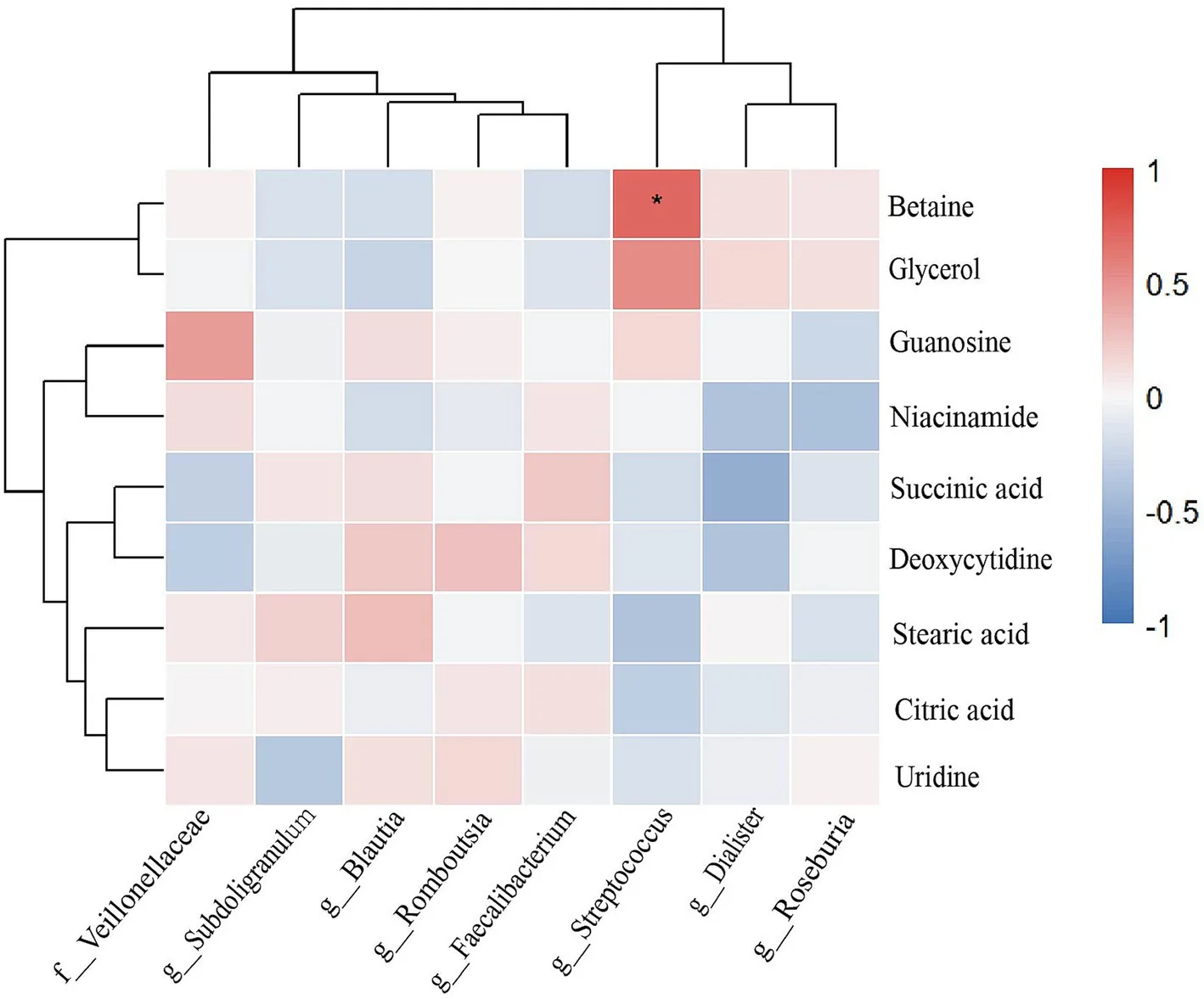
Associations between serum metabolites and differential gut microbial taxa in patients with hyperuricemia and uric acid nephropathy. Spearman’s rank correlation analysis was used to assess the relationships between candidate serum metabolites and differential bacterial taxa in the HUA and UAN groups. The color scale represents Spearman’s correlation coefficients, with red and blue indicating positive and negative correlations, respectively. Taxonomic prefixes *g__* and *f__* denote the genus and family levels, respectively. HUA, hyperuricemia; UAN, uric acid nephropathy.

### 3.5 Candidate metabolic signatures associated with uric acid nephropathy

An exploratory integrative assessment of pathway context, microbiota–metabolite associations, and MIMOSA2-predicted microbial contributions was used to prioritize nine candidate metabolites for further analysis: citric acid, succinic acid, deoxycytidine, uridine, guanosine, betaine, nicotinamide, glycerol, and stearic acid. This panel was derived from exploratory multi-omics integration. Associations with clinical indices were subsequently evaluated using *post hoc* correlation analyses and were not used for candidate selection.

Based on their biochemical properties and enriched pathways, these metabolites were grouped into four functional modules. The first module comprised short-chain fatty acid- and tricarboxylic acid cycle-related metabolism, represented by citric acid (C00158) and succinic acid (C00042). The second module was nucleoside metabolism, including deoxycytidine (C00881), uridine (C00299), and guanosine (C00387). The third module was related to inflammation-associated metabolism and included betaine (C00719) and nicotinamide (C00153). The fourth module involved lipid metabolism, represented by glycerol (C00116) and stearic acid (C01530).

Taken together, these nine candidate metabolites capture multiple biological dimensions associated with UAN, including microbial dysbiosis, metabolic perturbation, and renal functional impairment. Among them, citric acid, succinic acid, deoxycytidine, and uridine exhibited broad predicted microbial contribution profiles and close clinical associations, supporting their prioritization as exploratory metabolic signatures of hyperuricemia-related renal involvement.

### 3.6 Exploratory discriminatory performance of selected metabolic and microbial signatures

Exploratory ROC analyses were restricted to the HUA and UAN groups because the objective was to assess discrimination of renal involvement among patients with hyperuricemia. Metabolite-based analyses included 20 patients (HUA, *n* = 10; UAN, *n* = 10), whereas bacterial genus–based analyses included 62 patients (HUA, *n* = 30; UAN, *n* = 32). Among the individual features, *Streptococcus* showed the highest AUC (0.739, 95% CI, 0.613–0.864). For direct comparison, the multivariable models were evaluated in the same paired-data subset of 20 patients (HUA, *n* = 10; UAN, *n* = 10). The internally cross-validated metabolic, microbial, and integrated models yielded AUCs of 0.52 (95% CI, 0.24–0.80), 0.71 (95% CI, 0.46–0.90), and 0.66 (95% CI, 0.40–0.90), respectively. The integrated model did not improve discrimination beyond the microbial model in the present exploratory cohort. Full ROC curves and performance metrics, including optimal cutoffs, sensitivity, and specificity, are provided in Supplementary Figure 9, Supplementary Table 2, respectively.

## 4 Discussion

By integrating clinical phenotyping, gut microbial profiling, serum untargeted metabolomics, and exploratory multi-omics analyses, this study provides a multidimensional view of renal involvement in hyperuricemia. Three principal findings warrant emphasis. First, compared with uncomplicated HUA, UAN was characterized by impaired renal function and a less favorable lipid profile despite comparable circulating uric acid concentrations. Second, the microbial alterations were selective rather than community-wide, with no significant differences in α-diversity or overall β-diversity but with changes in several specific taxa. Third, UAN was accompanied by broader serum metabolic remodeling involving central carbon, nucleotide, redox-related, and lipid metabolism. Integration of these datasets highlighted nine candidate metabolites associated with renal involvement; however, the observed microbial–metabolite relationships and predicted microbial contributions should be regarded as exploratory and hypothesis-generating rather than as evidence of causal microbial activity.

The clinical findings suggest that established renal involvement in hyperuricemia cannot be adequately characterized by serum uric acid concentration alone. Patients with UAN had higher serum creatinine, blood urea nitrogen, urinary albumin-to-creatinine ratio, total cholesterol, and low-density lipoprotein cholesterol, together with lower eGFR, whereas serum uric acid concentrations were comparable between the two patient groups. Circulating uric acid reflects a dynamic balance among production, renal and intestinal excretion, treatment exposure, and tissue distribution; consequently, a single serum measurement may not fully capture the cumulative urate-related burden relevant to renal injury. Experimental evidence also suggests that intestinal urate transport, epithelial barrier function, and microbial composition may influence urate handling and renal exposure through pathways involving transporters such as ABCG2, URAT1, GLUT9, and OAT1 (Cui et al., 2025; Cui et al., 2026; Wang H. et al., 2025; Ye et al., 2024). These mechanisms provide a biologically plausible context for the observed dissociation between circulating uric acid concentration and the renal phenotype.

Age and total cholesterol were independently associated with lower eGFR, further supporting the relevance of aging and lipid metabolism to the UAN phenotype. Age-related reductions in renal functional reserve and repair capacity may increase susceptibility to metabolic and inflammatory stress, whereas dysregulated lipid handling may contribute to tubular lipotoxicity and fibrosis. By contrast, BMI was positively associated with eGFR in the multivariable model. This finding should not be interpreted as evidence that greater adiposity protects renal function, because BMI is an imprecise surrogate of body composition and may also reflect differences in nutritional status, muscle mass, or disease-related weight change. Overall, the clinical findings are more consistent with an interrelated renal–metabolic phenotype than with a simple linear relationship between circulating uric acid and renal impairment.

The microbiome findings similarly argue against characterizing UAN as a state of generalized intestinal dysbiosis. Microbial α-diversity did not differ significantly among groups, and the substantial overlap observed in the PCoA and NMDS analyses, together with the nonsignificant Adonis test, indicated that the overall community structure was largely preserved. Instead, the principal microbial signals involved selected taxa. *Faecalibacterium* was more abundant in healthy controls, whereas *Streptococcus* was associated predominantly with HUA and *Dialister* showed higher abundance in UAN. The enrichment of *Faecalibacterium* in healthy controls is biologically plausible because this genus includes major butyrate-producing organisms involved in epithelial barrier maintenance and immune homeostasis, and reduced *Faecalibacterium* abundance has been reported in kidney disease (Hao et al., 2021; Li et al., 2022). Nevertheless, taxonomic abundance alone cannot establish altered butyrate production. Similar caution is warranted when interpreting *Lactobacillales* and *Streptococcus*, because metabolic functions may vary substantially across species and strains despite experimental evidence for urate-modulating effects of selected *Lactobacillus* strains (Xue et al., 2024). The association of *Dialister* with UAN should likewise be interpreted cautiously. Previous studies have linked *Dialister* to kidney-related microbial–metabolite networks, including amino acid metabolism (Elmorsy et al., 2025), but its enrichment in UAN does not establish a direct pathogenic role.

Sensitivity analyses showed that the directions of the *Streptococcus* and *Dialister* associations were preserved after adjustment for BMI and lipid-lowering medication use, with similar patterns observed among participants not receiving lipid-lowering therapy. However, neither taxonomic association remained significant after FDR correction. BMI-adjusted KEGG pathway and KO associations were likewise attenuated and did not survive FDR correction. These findings suggest that the observed microbial differences were not solely attributable to measured BMI or lipid-lowering therapy, although residual confounding cannot be excluded. Accordingly, these associations should be interpreted as exploratory and validated in larger cohorts.

Among the nine prioritized metabolites, citrate and succinate place central carbon metabolism at the intersection of several of these processes. Both are intermediates of the tricarboxylic acid cycle and may also be indirectly influenced by microbial fermentation networks. Their alterations could therefore reflect host mitochondrial dysfunction, changes in microbial substrate utilization, impaired renal handling, or a combination of these processes. Importantly, disturbances in TCA-cycle intermediates have also been reported in diabetic kidney disease, membranous nephropathy, IgA nephropathy, and other renal disorders (Danilova et al., 2023; Liu P. et al., 2025; Qu et al., 2024). Metabolomic patterns may also vary according to disease phenotype and biological matrix (Yeo et al., 2024). Citrate and succinate should therefore be viewed as components of a broader metabolic response associated with renal dysfunction in the setting of hyperuricemia rather than as UAN-specific biomarkers. Although extracellular succinate can participate in SUCNR1-mediated inflammatory and fibrotic signaling in experimental systems (Louis and Flint, 2017), the present findings alone do not establish activation of this pathway in UAN.

Alterations in uridine, deoxycytidine, and guanosine further suggest that nucleotide metabolism may represent another component of this broader metabolic phenotype. Because both purine and pyrimidine nucleosides were involved, the observed pattern is unlikely to simply reflect increased substrate availability for uric acid synthesis. Instead, it may reflect coordinated changes in nucleotide synthesis, salvage, cellular turnover, energy metabolism, microbial utilization, or renal handling (Kim et al., 2024; Tu et al., 2025; Weng et al., 2018). Likewise, changes in nicotinamide and betaine may reflect alterations in redox, one-carbon, and stress-response metabolism. Nicotinamide participates in NAD-related pathways involved in mitochondrial function, DNA repair, and cellular stress responses (Curran and Kopp, 2023; Yang et al., 2024), whereas betaine has recognized roles as an osmolyte and methyl-group donor, with antioxidant and anti-inflammatory properties (Rasineni et al., 2026; Zawieja and Chmurzynska, 2025). These metabolite changes may therefore reflect altered utilization, compensatory responses, or systemic responses to renal and metabolic stress rather than a single pathogenic process.

The lipid-related findings further suggest that conventional lipid abnormalities and metabolite-level lipid remodeling capture different biological dimensions. Clinically, UAN was associated with a higher prevalence of hyperlipidemia and higher total and LDL cholesterol concentrations, whereas glycerol and stearic acid showed distinct metabolomic patterns. Conventional lipid measurements reflect aggregate circulating lipid pools, whereas individual metabolites may capture processes such as lipolysis, cellular uptake, fatty acid oxidation, membrane remodeling, and substrate redistribution. Previous lipidomic studies have demonstrated extensive alterations in glycerolipid and glycerophospholipid metabolism in hyperuricemia (Kang et al., 2024; Xiao et al., 2024; Zou et al., 2025). Thus, the present findings are consistent with broader lipid remodeling rather than directly indicating lipid depletion or excess at the tissue level.

The integrative analyses were intended to place these metabolic alterations within a microbial and clinical context rather than to demonstrate a mechanistic gut–kidney pathway. Correlations between selected taxa, metabolites, and renal indices indicate statistical co-variation but do not establish directionality. Similarly, MIMOSA2 estimates microbial metabolic potential from community composition and reference metabolic networks. The broad predicted contribution profiles observed for citrate, deoxycytidine, succinate, and uridine are therefore consistent with a possible microbial component of these metabolic patterns, but they do not demonstrate direct microbial production, metabolic flux, or a dominant microbial origin of circulating metabolites. Serum metabolite concentrations integrate the combined effects of microbial synthesis and consumption, intestinal absorption, host production, tissue utilization, dietary and medication exposure, and renal clearance. The nine-metabolite panel is therefore better interpreted as an exploratory signature of integrated host–microbial metabolic remodeling associated with renal involvement rather than as a direct measure of microbial activity.

This distinction is also relevant to the exploratory ROC analyses. Although *Streptococcus* showed the highest AUC among the individual features, the internally cross-validated multivariable models demonstrated only modest discriminatory performance, and integration of microbial and metabolic features did not improve performance beyond the microbial model alone. Thus, biological relevance should not be equated with discriminatory performance. The present signatures may help characterize the biological context of renal involvement in hyperuricemia, but they should not be regarded as validated diagnostic biomarkers. Their incremental value beyond established clinical markers will require evaluation in larger independent cohorts and direct comparison with other chronic kidney disease etiologies.

Several features strengthen the present study. The clinical cohort allowed the molecular findings to be interpreted against well-characterized renal and metabolic phenotypes, while the multi-omics component enabled microbial, metabolic, and clinical information to be considered within a common analytical framework. Sensitivity analyses addressing BMI and major medication exposures also helped assess the robustness of selected microbial associations. More broadly, the integration of taxonomic, metabolomic, and clinical data provided complementary perspectives on the biological context of renal involvement in hyperuricemia.

Taken together, the present findings suggest that renal involvement in hyperuricemia is accompanied by a coordinated clinical and molecular phenotype extending beyond circulating uric acid concentration alone. This phenotype comprises renal dysfunction, lipid metabolic abnormalities, selective microbial alterations, and changes in central carbon, nucleotide, redox-related, and lipid metabolism. The nine candidate metabolites therefore provide an exploratory molecular framework for characterizing the metabolic context of UAN rather than a demonstrated mechanistic pathway or validated diagnostic panel.

## 5 Limitations

Several limitations should be acknowledged. First, the cross-sectional design precludes causal inference, and the modest sample size, particularly the small HC group, limits the precision and generalizability of the multi-omics findings; HC-based analyses were therefore considered exploratory. Second, functional inference based on 16S rRNA gene sequencing and MIMOSA2 is constrained by the limited species- and strain-level resolution of 16S data and relies on reference genome-based metabolic annotations rather than direct measurement of microbial gene content or activity. Accordingly, the inferred microbial contributions should be regarded as predictive rather than direct functional evidence and require validation using higher-resolution approaches, such as shotgun metagenomics, together with targeted metabolomic analyses. Residual confounding also remains possible. Although sensitivity analyses adjusted for BMI and major medication exposures, treatment patterns differed between the HUA and UAN groups, and habitual dietary intake was not quantitatively assessed; therefore, confounding related to medication indication, treatment characteristics, diet, comorbidities, renal clearance, and baseline metabolic status cannot be fully excluded. Third, the proposed signatures were derived and internally evaluated within the same cohort and require external validation. Finally, the absence of disease-control groups with other CKD etiologies limits assessment of disease specificity, particularly because alterations in citrate, succinate, and other TCA-cycle intermediates may reflect shared renal and metabolic disturbances. Larger prospective studies incorporating standardized dietary and medication assessment, external validation, and appropriate disease controls are needed to confirm the specificity and clinical relevance of these findings.

## 6 Conclusion

Our integrated clinical, gut microbiome, and serum metabolomic analyses identified distinct microbial alterations and metabolic perturbations associated with UAN compared with uncomplicated HUA. The nine candidate metabolites provide an exploratory molecular framework linking renal dysfunction to host–microbial metabolic dysregulation. However, the selected signatures showed only modest discriminatory performance and should not be considered validated diagnostic biomarkers. Further validation using targeted quantitative assays in larger independent prospective cohorts is warranted to establish their biological and clinical relevance.

## Data Availability

The datasets presented in this study can be found in online repositories. The names of the repository/repositories and accession number(s) can be found at: https://www.ncbi.nlm.nih.gov/, BioProject accession number: PRJNA1497093.
