# Supplementary material for: Integrated gut microbiome and serum metabolomics identify candidate metabolic signatures of uric acid nephropathy
Source: Front Microbiol. 2026 Oct 8;17:1941602. doi: 10.3389/fmicb.2026.1941602 (PMC13687322; doi:10.3389/fmicb.2026.1941602)
Supplement: Supplementary file 1 [file Presentation_1.PDF]

Supplementary Material

1 Supplementary Figures

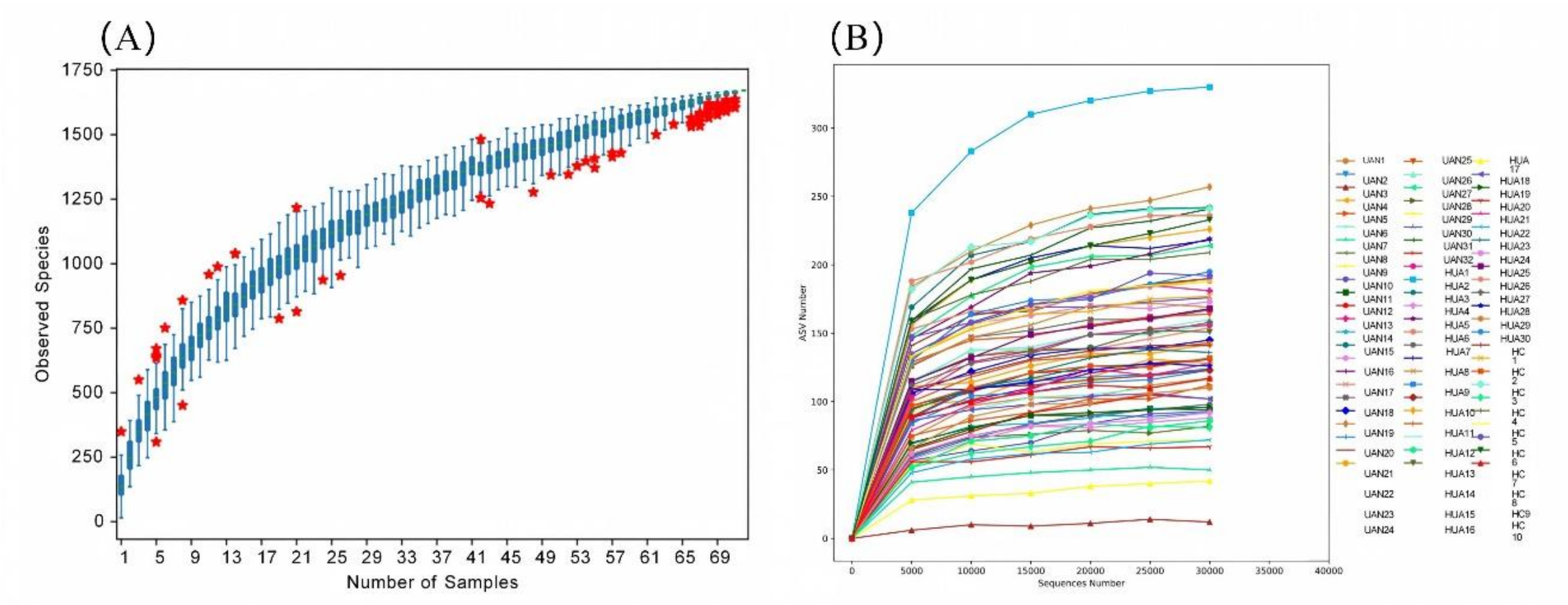

Supplementary Figure 1. Sequencing depth and sampling adequacy assessment of gut microbiota.（A） Species accumulation curve based on ASVs; (B) rarefaction curves of ASVs across samples. The plateauing trends indicate that the sequencing depth and sampling effortwere sufficient to capture the major microbial diversity for downstream analysis. ASV, amplicon sequence variant.

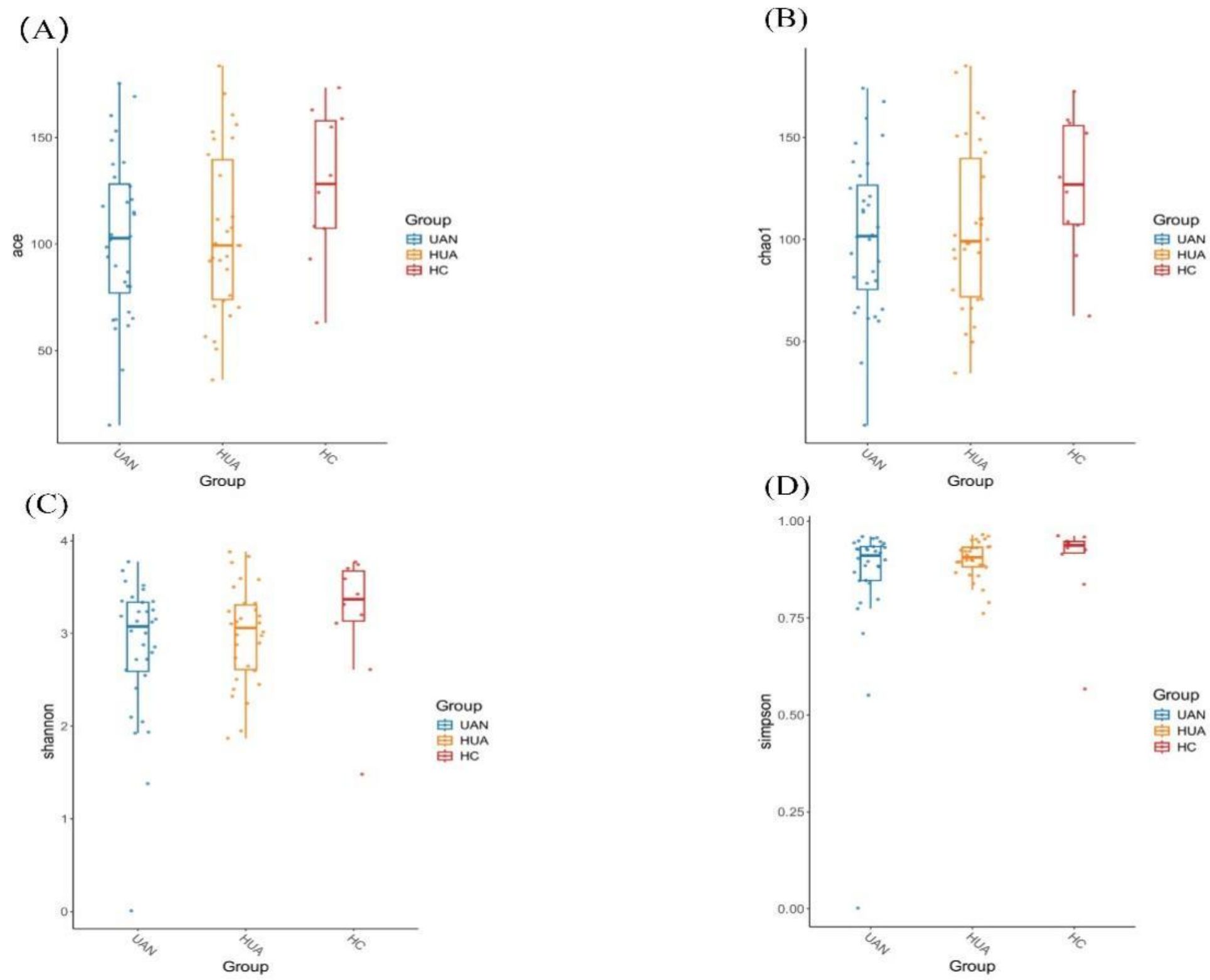

Supplementary Figure 2. Gut microbiota alpha diversity among HC, HUA, and UAN. Boxplots showing alpha diversity indices, including ACE (A)Chao1 (B), Shannon (C), and Simpson (D). Each dot represents an individual sample. Abbreviations: HC, healthy control; HUA, hyperuricemia; UAN, uric acid nephropathy; ACE, abundance-based coverage estimator.

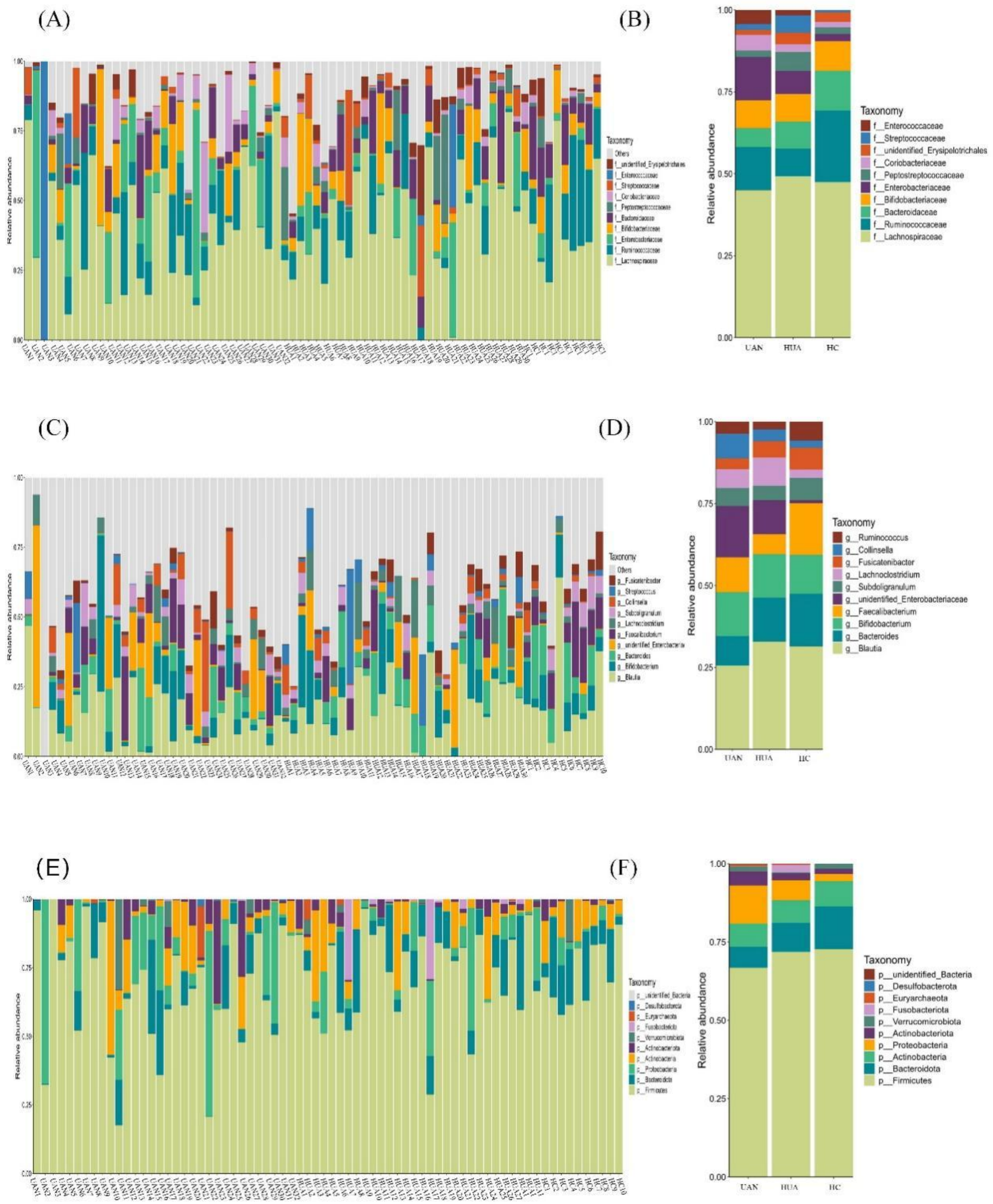

Supplementary Figure 3. Taxonomic profiles of gut microbiota among HC, HUA, and UAN. (A) Family-level taxonomic composition across individual samples; (B) family-level composition summarized by group; (C) genus-level taxonomic composition across individual samples; (D) genus-level composition summarized by group; E phylum-level taxonomic composition across individual samples; (F) phylum-level composition summarized by group. The relative abundance of dominant taxa is shown, with low-abundance taxa grouped as "Others". Abbreviations: HC, healthy control; HUA, hyperuricemia; UAN, uric acid nephropathy.

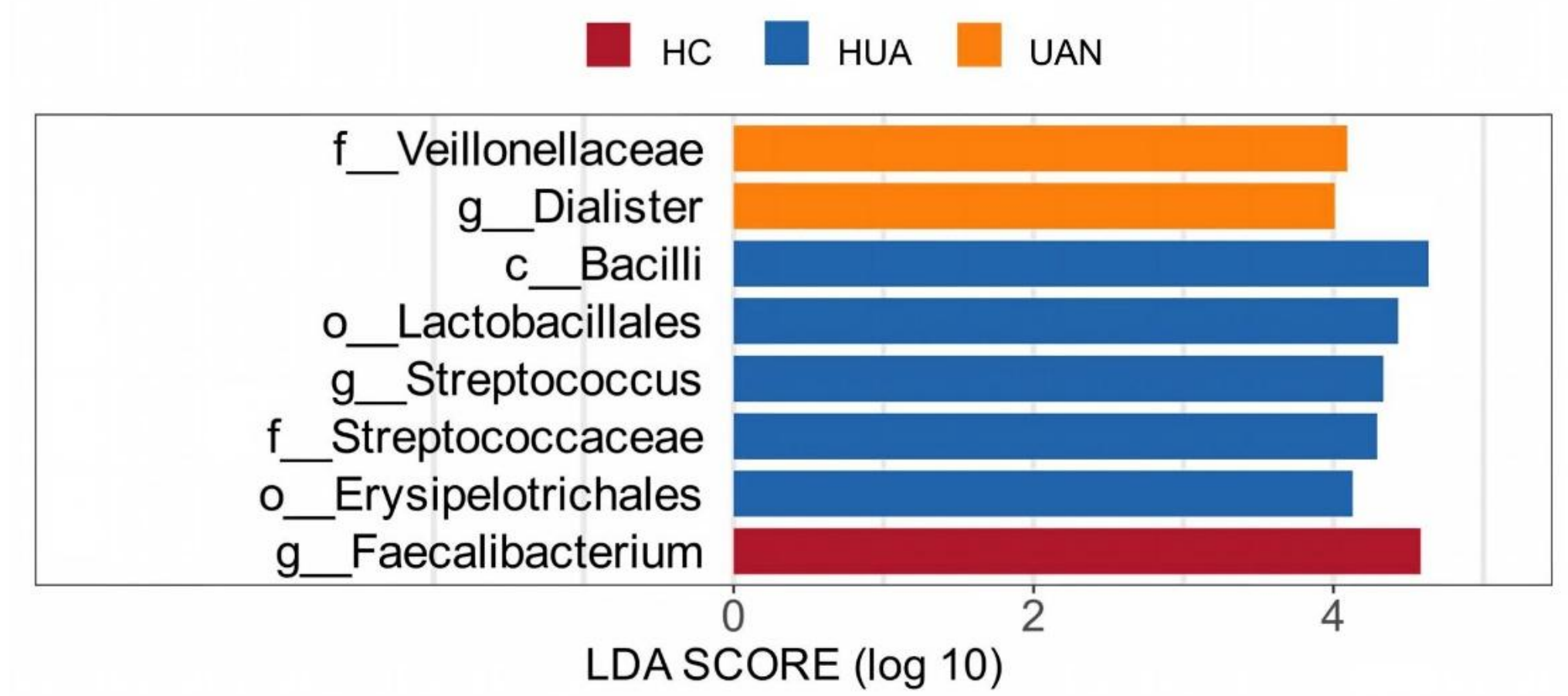

Supplementary Figure 4. Exploratory LEfSe analysis of gut microbial taxa across the HC, HUA, and UAN groups. LEfSe analysis with an LDA score threshold of ≥4.0 identified *Faecalibacterium* as HC-associated; Bacilli, Lactobacillales, Streptococcaceae, *Streptococcus*, and Erysipelotrichales as HUA-associated; and Veillonellaceae and *Dialister* as UAN-associated. Given the small HC sample size (n = 10), HC-related findings should be interpreted as exploratory.

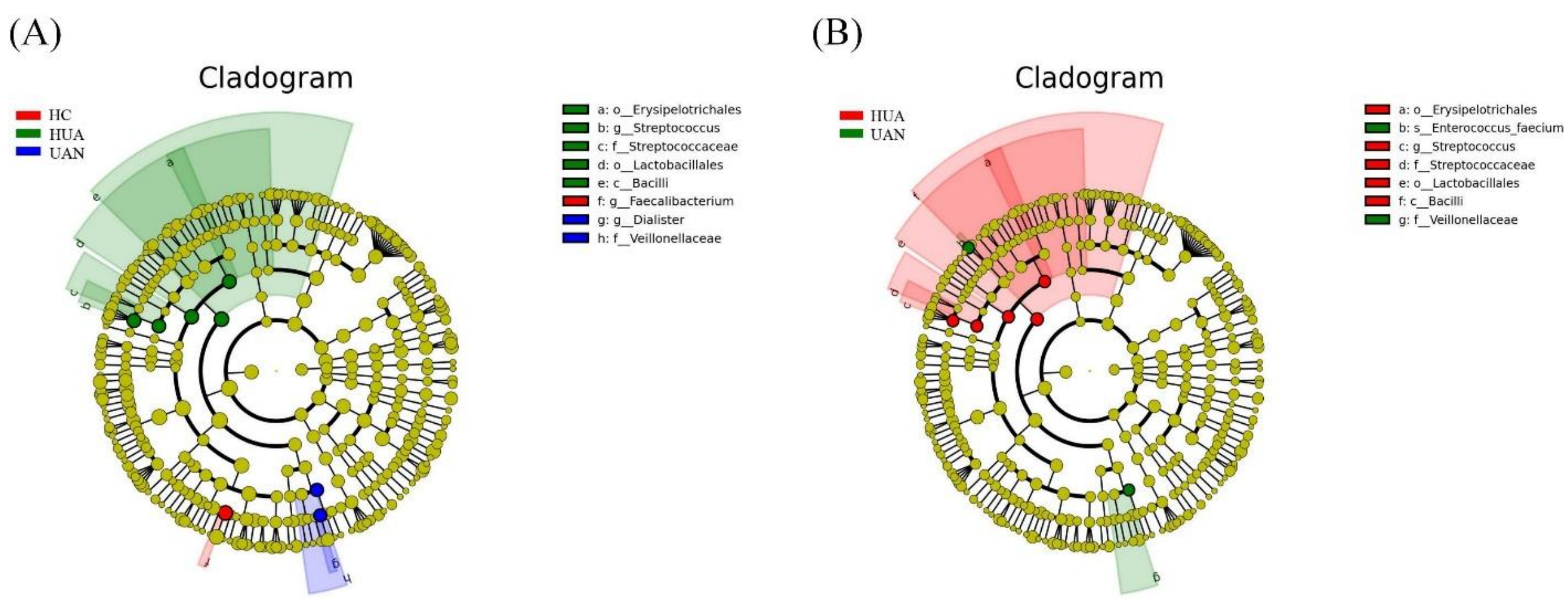

Supplementary Figure 5. LEfSe cladogram of differential gut microbiota among HC, HUA, and UAN.

(A) LEfSe cladogram showing the phylogenetic distribution of discriminative bacterial taxa among HC, HUA, and UAN;(B) LEfSe cladogram showing discriminative bacterial taxa between HUA and UAN. Colored nodes indicate taxa enriched in the corresponding groups, whereas yellow nodes represent taxa without group-specific enrichment. The cladograms display microbial taxa from higher to lower taxonomic levels. Abbreviations: HC, healthy control; HUA, hyperuricemia; UAN, uric acid nephropathy; LEfSe, linear discriminant analysis effect size.

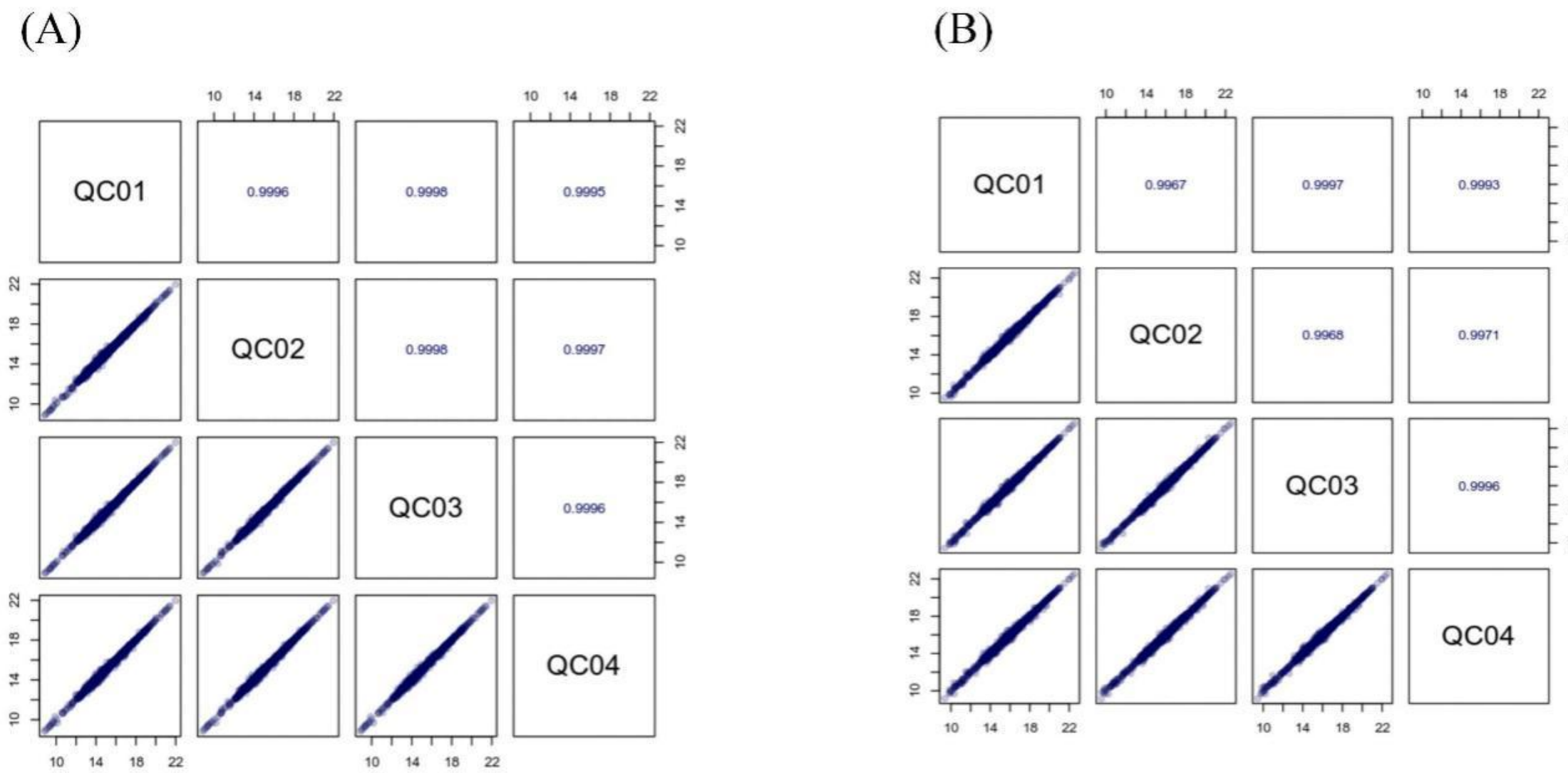

Supplementary Figure 6. Reproducibility evaluation of serum untargeted metabolomics data. **(A)** Pairwise correlation matrix of QC samples in the positive ion mode; (**B**) pairwise correlation matrix of QC samples in the negative ion mode. The high correlation coefficients among QC injections indicate stable instrumental performance and satisfactory data reproducibility. **Abbreviations:** QC, quality control.

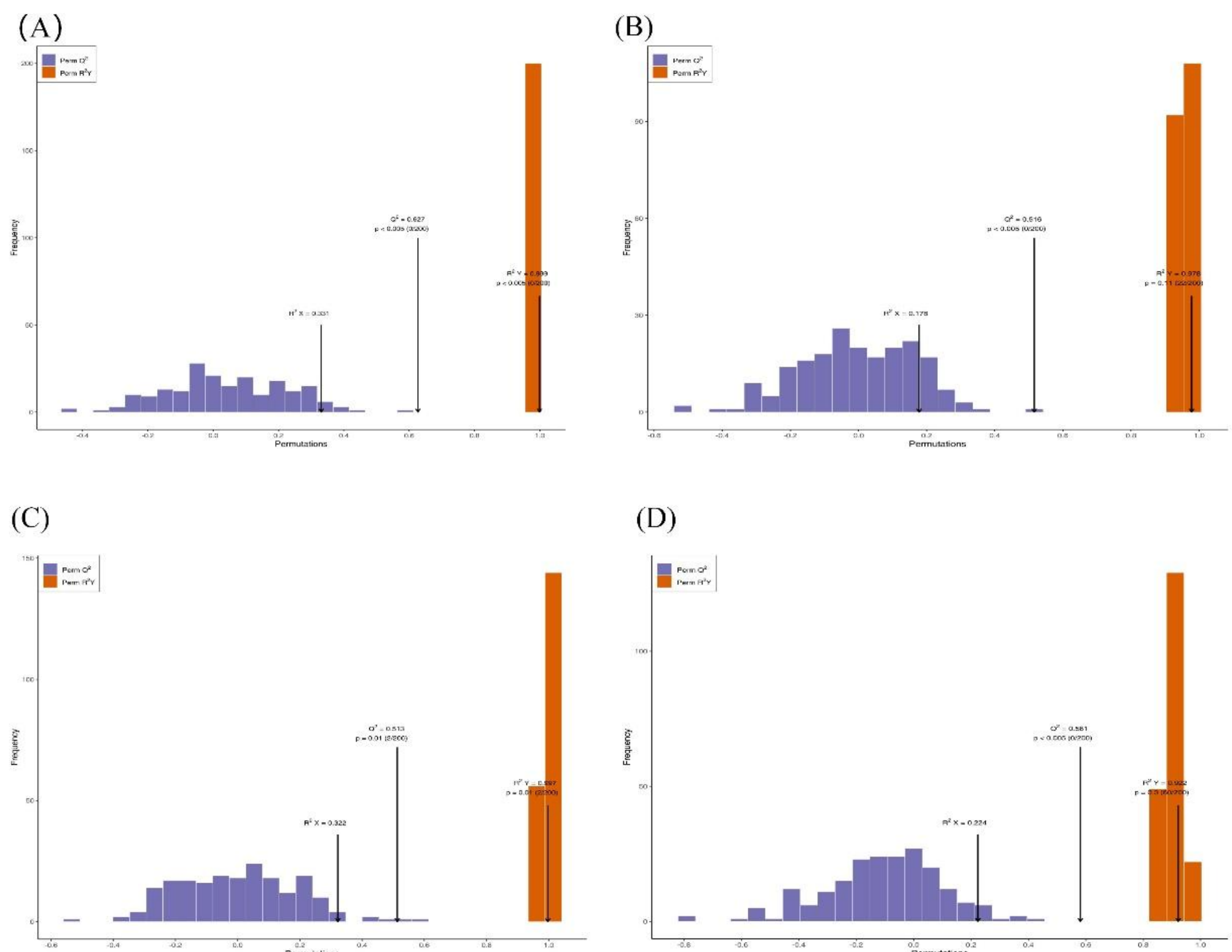

Supplementary Figure 7. OPLS-DA model validation by permutation testing.(A–D) Permutation tests of OPLS-DA models for HUA versus HC (A), UAN versus HUA (B), UAN versus HC (C), and HC/HUA/UAN (D). The models were validated using 200 random permutations to evaluate predictive reliability and the risk of overfitting. Abbreviations: HC, healthy control; HUA, hyperuricemia; UAN, uric acid nephropathy; OPLS-DA, orthogonal partial least squares discriminant analysis; R^2Y, explained response variation; Q^2, predictive ability.

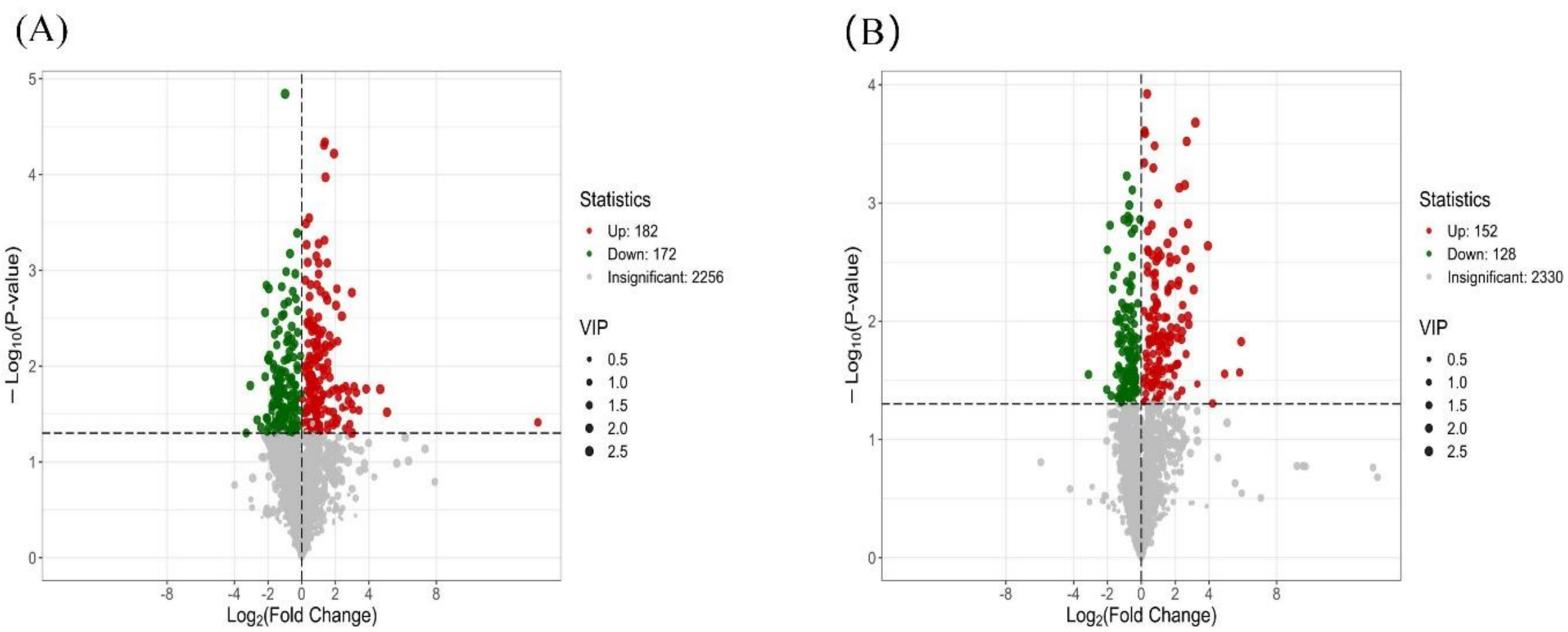

Supplementary Figure 8. Differential serum metabolite profiles in additional pairwise comparisons. (A) Volcano plot for HC versus HUA; (**B**) volcano plot for HC versus UAN. Red, green, and grey dots represent upregulated, downregulated, and non-significant metabolites, respectively. Dot size indicates the VIP score. Dashed lines denote the predefined thresholds for significance and fold change. **Abbreviations:** HC, healthy control; HUA, hyperuricemia; UAN, uric acid nephropathy; VIP, variable importance in projection.

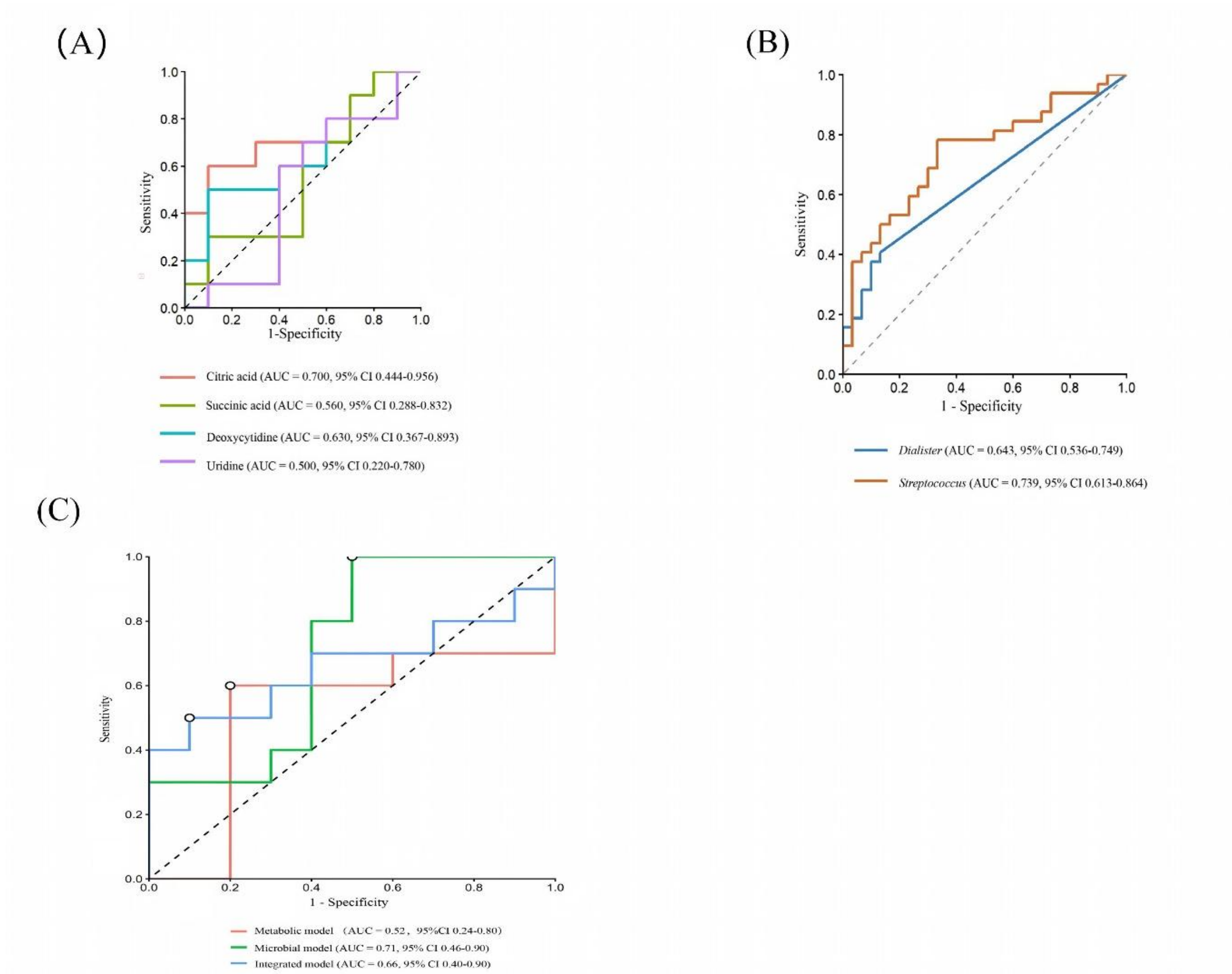

Supplementary Figure 9. Exploratory ROC analyses of selected metabolic and microbial signatures for distinguishing UAN from uncomplicated HUA. (A) ROC curves for citric acid, succinic acid, deoxycytidine, and uridine (HUA, n = 10; UAN, n = 10). (B) ROC curves for the differential bacterial genera *Dialister* and *Streptococcus* (HUA, n = 30; UAN, n = 32). (C) ROC curves for the metabolic, microbial, and integrated models evaluated in the same complete-case subset (HUA, n = 10; UAN, n = 10). The metabolic model included the four metabolites, the microbial model included *Dialister* and *Streptococcus*, and the integrated model combined all six features. Model-level ROC curves were based on out-of-fold predicted probabilities generated using repeated stratified 5-fold nested cross-validation. The diagonal dashed line denotes chance-level discrimination (AUC = 0.50). AUCs and 95% CIs are shown in the figure. **Abbreviations:** ROC, receiver operating characteristic; AUC, area under the curve; CI, confidence interval; HUA, hyperuricemia; UAN, uric acid nephropathy.

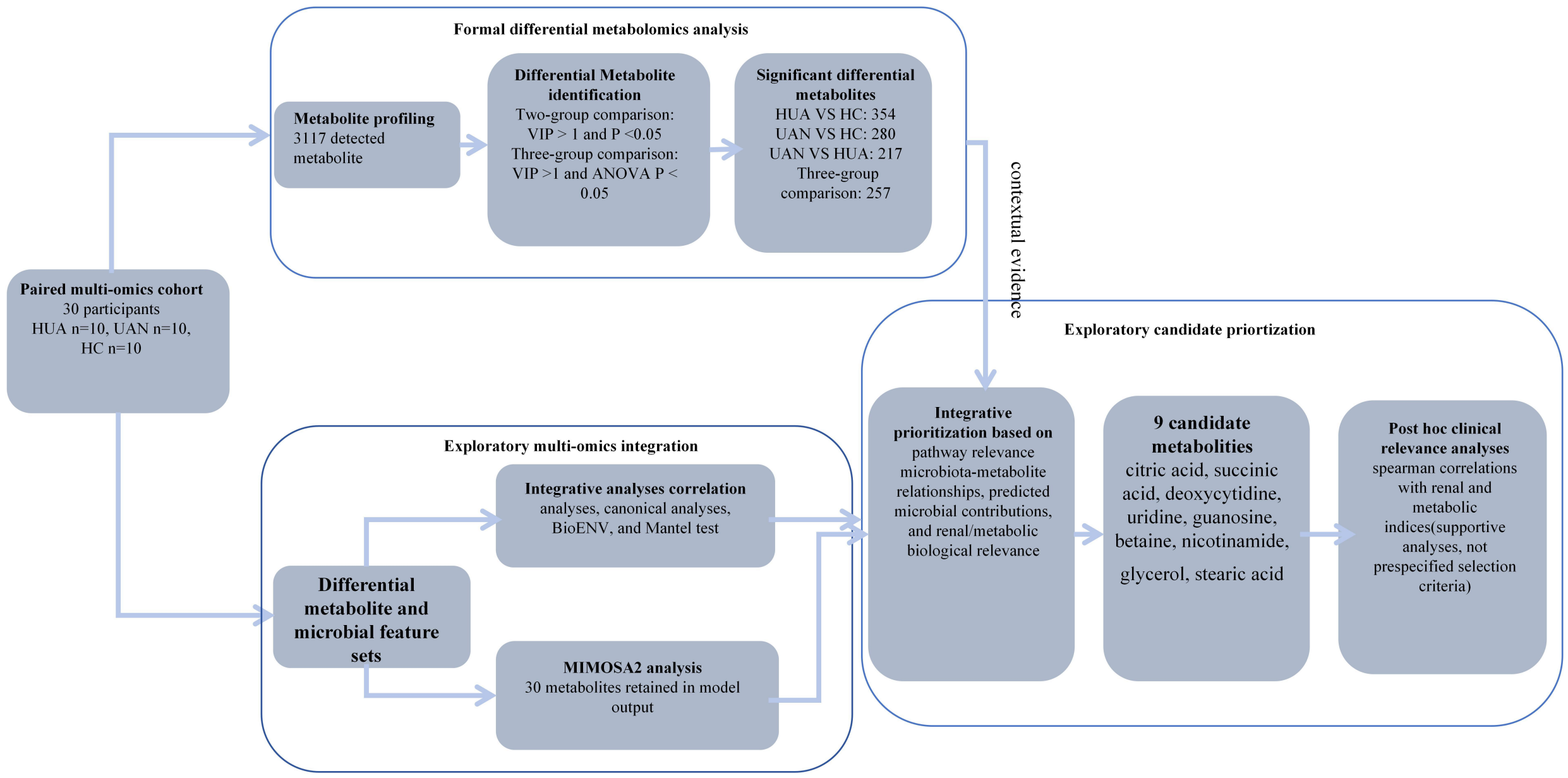

Supplementary Figure 10. Analytical workflow for differential metabolomics and exploratory prioritization of candidate metabolites. Paired metabolome–microbiome data from 30 participants (HUA, n = 10; UAN, n = 10; HC, n = 10) were analyzed. Differential metabolites were defined using VIP > 1.0 and $P < 0.05$ for two-group comparisons and VIP > 1.0 and ANOVA $P < 0.05$ for the three-group comparison, yielding 354 metabolites for HUA versus HC, 280 for UAN versus HC, 217 for UAN versus HUA, and 257 in the three-group comparison. Exploratory multi-omics integration incorporated metabolomic and microbial features, correlation and multivariate analyses, and MIMOSA2-predicted microbial contributions. Nine candidate metabolites were subsequently highlighted through integrative interpretation of pathway context, microbiota–metabolite relationships, predicted microbial contributions, and biological relevance. Post hoc clinical correlation analyses were performed as supportive analyses and were not used as prespecified selection criteria.

2 Supplementary Tables

Supplementary Table 1 Pairwise comparisons and effect sizes for selected bacterial genera

Genus	Comparison	n	Group 1, median (IQR)	Group 2, median (IQR)	Cliff's δ	95% CI	Wilcoxon P	BH-adjusted P
Dialister	UAN vs HUA	30 vs 32	0 (0–0.0072)	0 (0–0)	0.285	0.060–0.483	0.014	-
Faecalibacterium	HC vs HUA	10 vs 30	0.1067 (0.0585–0.1271)	0.0058 (0.0001–0.0211)	0.620	0.239–0.836	0.004	0.008
Faecalibacterium	HC vs UAN	10 vs 32	0.1067 (0.0585–0.1271)	0.0160 (0.0008–0.0958)	0.406	0.027–0.683	0.057	0.057

Note: Data are presented as median (interquartile range [IQR]) relative abundance. Pairwise comparisons were performed using the Wilcoxon rank-sum test, with Cliff's δ and 95% confidence intervals (CIs) reported as distribution-free effect-size estimates. For *Faecalibacterium*, positive Cliff's δ values indicate higher abundance in HC than in the corresponding patient group; P values for the two HC-based comparisons were adjusted using the Benjamini–Hochberg (BH) procedure. Given the small HC sample size (n = 10), these comparisons were considered exploratory. For *Dialister*, Cliff's δ was calculated for UAN versus HUA, with positive values indicating higher abundance in UAN. The 95% CIs for Cliff's δ were not multiplicity-adjusted; multiplicity across the two HC-based comparisons was controlled using Benjamini–Hochberg-adjusted P values.
Abbreviations: HC, healthy controls; HUA, hyperuricemia; UAN, uric acid nephropathy; CI, confidence interval; IQR, interquartile range.

Supplementary Table 2 Exploratory discriminatory performance of selected metabolic and microbial features and multivariable models for distinguishing uric acid nephropathy from uncomplicated hyperuricemia

Feature/model	AUC	95% CI	Optimal cutoff	Sensitivity, %	Specificity, %
**Citric acid**	0.7	（0.444，0.956）	79848693.32	60	90
**Succinic acid**	0.56	（0.288，0.832）	20699099.74	100	20
**Deoxycytidine**	0.63	（0.367，0.893）	55964323.12	50	90
**Uridine**	0.5	（0.22，0.78）	8473284.385	80	40
**Dialister**	0.643	（0.536，0.749）	0.000222	37.5	90

**Streptococcus**	0.739	（0.613，0.864）	0.004633	78.1	66.7
**Metabolic model**	0.52	（0.24，0.8）	0.8	60	80
**Microbial model**	0.71	（0.46，0.90）	0.8	100	50
**Integrated model**	0.66	（0.40，0.90）	0.9	50	90

Note: AUCs are presented with 95% confidence intervals (CIs). Optimal cutoff values were determined by maximizing the Youden index. For serum metabolites, cutoff values correspond to processed LC–MS peak intensities (arbitrary units) and should not be interpreted as absolute concentration thresholds. For bacterial genera, cutoff values represent relative abundances. For the multivariable metabolic, microbial, and integrated models, ROC analysis and optimal cutoff determination were based on out-of-fold predicted probabilities generated using repeated stratified 5-fold nested cross-validation. Sensitivity and specificity were calculated at the corresponding optimal cutoff. These analyses were exploratory and were not externally validated. **Abbreviations:** AUC, area under the receiver operating characteristic curve; CI, confidence interval; LC–MS, liquid chromatography–mass spectrometry; HUA, hyperuricemia; UAN, uric acid nephropathy; ROC, receiver operating characteristic.

Supplementary Table 3. BMI-adjusted associations between disease status and genus-level gut microbial features in patients with HUA and UAN. Provided as a separate Excel file.

Supplementary Table 4. BMI-adjusted associations between disease status and predicted KEGG pathway profiles in patients with HUA and UAN. Provided as a separate Excel file.

Supplementary Table 5. Supplementary Table 5. Medication use and sensitivity analyses for key bacterial genera in the HUA and UAN groups. Panel A. Medication use at biospecimen collection; Panel B. Sensitivity analyses for key bacterial genera

Supplementary Data 1. Complete list of annotated serum metabolites retained after quality-control and annotation filtering. The complete metabolite annotation dataset is provided as a separate Excel file. The nine metabolites prioritized in the subsequent exploratory multi-omics integration are indicated in the final column for traceability.

Supplementary Data 2. Complete BMI-adjusted associations between disease status and predicted KEGG Orthology profiles in patients with HUA and UAN. Provided as a separate Excel file.

Supplementary Data 3. Supplementary Data 3. Complete genus-level sensitivity analyses after adjustment for BMI and lipid-lowering medication use in patients with HUA and UAN. Provided as a separate Excel file.
